# Accuracy of Five Algorithms to Diagnose Gambiense Human African Trypanosomiasis

**DOI:** 10.1371/journal.pntd.0001233

**Published:** 2011-07-05

**Authors:** Francesco Checchi, François Chappuis, Unni Karunakara, Gerardo Priotto, Daniel Chandramohan

**Affiliations:** 1 Faculty of Infectious and Tropical Diseases, London School of Hygiene and Tropical Medicine, London, United Kingdom; 2 Médecins Sans Frontières, Geneva, Switzerland; 3 Geneva University Hospitals and University of Geneva, Geneva, Switzerland; 4 Mailman School of Public Health, Columbia University, New York, New York, United States of America; 5 Epicentre, Paris, France; Foundation for Innovative New Diagnostics (FIND), Switzerland

## Abstract

**Background:**

Algorithms to diagnose gambiense human African trypanosomiasis (HAT, sleeping sickness) are often complex due to the unsatisfactory sensitivity and/or specificity of available tests, and typically include a screening (serological), confirmation (parasitological) and staging component. There is insufficient evidence on the relative accuracy of these algorithms. This paper presents estimates of the accuracy of five algorithms used by past Médecins Sans Frontières programmes in the Republic of Congo, Southern Sudan and Uganda.

**Methodology and Principal Findings:**

The sequence of tests in each algorithm was programmed into a probabilistic model, informed by distributions of the sensitivity, specificity and staging accuracy of each test, constructed based on a literature review. The accuracy of algorithms was estimated in a baseline scenario and in a worst-case scenario introducing various near worst-case assumptions. In the baseline scenario, sensitivity was estimated as 85–90% in all but one algorithm, with specificity above 99.9% except for the Republic of Congo, where CATT serology was used as independent confirmation test: here, positive predictive value (PPV) was estimated at <50% in realistic active screening prevalence scenarios. Furthermore, most algorithms misclassified about one third of true stage 1 cases as stage 2, and about 10% of true stage 2 cases as stage 1. In the worst-case scenario, sensitivity was 75–90% and PPV no more than 75% at 1% prevalence, with about half of stage 1 cases misclassified as stage 2.

**Conclusions:**

Published evidence on the accuracy of widely used tests is scanty. Algorithms should carefully weigh the use of serology alone for confirmation, and could enhance sensitivity through serological suspect follow-up and repeat parasitology. Better evidence on the frequency of low-parasitaemia infections is needed. Simulation studies should guide the tailoring of algorithms to specific scenarios of HAT prevalence and availability of control tools.

## Author Summary

Gambiense human African trypanosomiasis (HAT, sleeping sickness) usually features low prevalence. The two stages of the disease require different treatments, and stage 2 is fatal if untreated. HAT diagnosis must therefore be highly sensitive (i.e., detect as many true cases as possible) and specific (i.e., minimize false positives). HAT diagnostic algorithms are complex and involve several tests to screen for, confirm and stage infection. We analyzed five algorithms used by Médecins Sans Frontières HAT programmes. We combined published data on the accuracy of each test in the algorithm with a computer program that simulates all possible algorithm branches. We found that all algorithms had reasonable sensitivity (85–90%); specificity was high (>99.9%) except for the Republic of Congo, where confirmation did not rely on microscopic evidence, resulting in frequent false positives (but also higher sensitivity). Algorithms misclassified about one third of stage 1 cases as stage 2, but stage 2 classification was highly accurate. The use of serology alone for confirmation merits caution. HAT diagnosis could be made more sensitively by following up serological suspects and repeating microscopic examinations. Computer simulations can help to adapt algorithms to local conditions in each HAT programme, such as the prevalence of infection and operational constraints.

## Introduction

The diagnosis of gambiense human African trypanosomiasis (HAT, sleeping sickness) in routine conditions is complex [1]. Because infection prevalence is usually low (<1–2%), diagnostic tests require a high sensitivity and specificity to achieve adequate positive predictive value (PPV). Furthermore, accurate classification into stage 1 (haemo-lymphatic) and 2 (meningo-encephalitic) is crucial: the stage 1 treatment, pentamidine, is inefficacious for stage 2 due to limited blood brain barrier penetration [2], while, of the two stage 2 treatments, melarsoprol is highly toxic [3] and eflornithine-nifurtimox is cumbersome to administer.

No single HAT diagnostic test currently offers satisfactory sensitivity and specificity. Diagnostic algorithms therefore combine several tests and feature a screening, confirmation and staging component. The Card Agglutination Test for Trypanosomiasis (CATT) [4], highly sensitive when performed in whole blood (CATT-wb) but insufficiently specific (<96%), is used for screening. After CATT-wb or CATT plasma screening, various parasitological confirmation tests are applied either alone or in sequence on blood and/or neck gland aspirate, so as to maximise specificity while maintaining acceptable levels of sensitivity. Various dilutions of the CATT in plasma (between 1∶4 and 1∶16) may also be performed ahead of parasitology to reduce the number of individuals needing parasitological testing. Parasitological positives (T+) undergo lumbar puncture and are classified as stage 2 if parasites are found in cerebrospinal fluid (CSF), or if a given threshold of CSF white blood cell (WBC) density (ranging from 5 to 20/µL) is exceeded [5]. Individuals with strong CATT reactions (dilutions ≥1∶4) but no parasitological evidence of infection (T−) are generally considered serological suspects. Some control programmes follow-up suspects for up to one year, repeating parasitological tests. Others consider them non-cases or treat them presumptively. The underlying infection prevalence affects the relative efficiency of these different strategies [6], [7], [8].

The accuracy of HAT diagnostic algorithms has not been documented in detail, partly because their complexity precludes straightforward analysis. Here, we present estimates of the accuracy of five different diagnostic algorithms used by Médecins Sans Frontières (MSF) in past gambiense HAT control programmes using summary estimates of reported accuracy of individual HAT tests and a probabilistic model.

## Methods

### Description of the MSF algorithms

**Figure 1:**
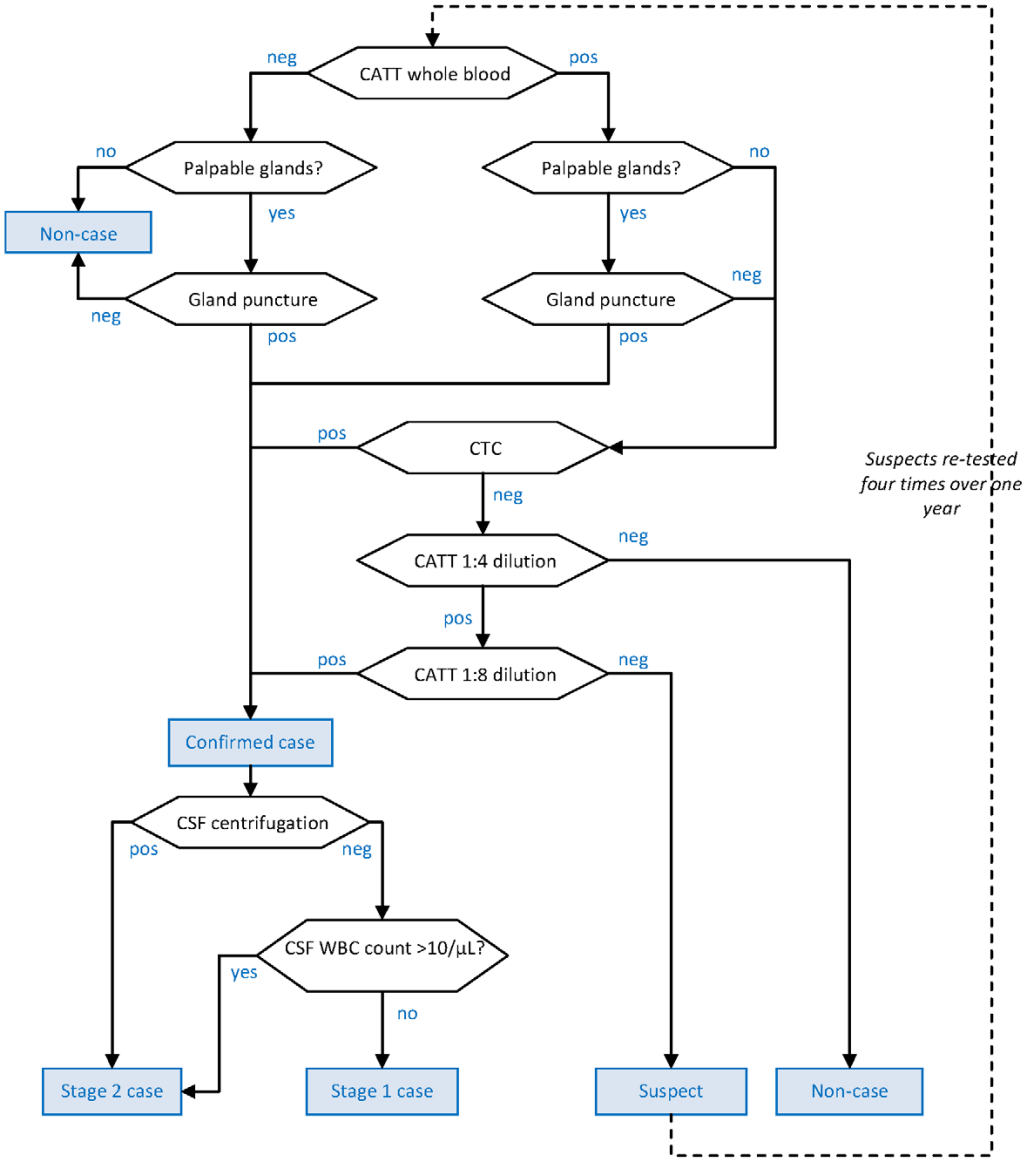
Diagnostic algorithm used in the Gamboma, Mossaka and Nkayi, Republic of Congo programmes. Hexagonal boxes indicate tests. Square, blue-shaded boxes indicate points at which a decision on the patient is reached.

The five algorithms (shown in Figures 1 to [Fig pntd-0001233-g002] [Fig pntd-0001233-g003] [Fig pntd-0001233-g004] 5) were used in projects in the Republic of Congo (Gamboma, Plateaux Region, 2001–2003; Mossaka, Cuvette Region, 2003–2005; Nkayi, Bouenza Region, 2003–2005); Southern Sudan (Kiri, Kajo Keji County, Central Equatoria, 2000–2007); and Uganda (Adjumani District, 1991–1996; Arua and Yumbe Districts, 1995–2002). The Southern Sudan project made progressive modifications to its algorithm, but only the first (old) and the last (new) algorithms used by that project are assessed here.

**Figure 2 pntd-0001233-g002:**
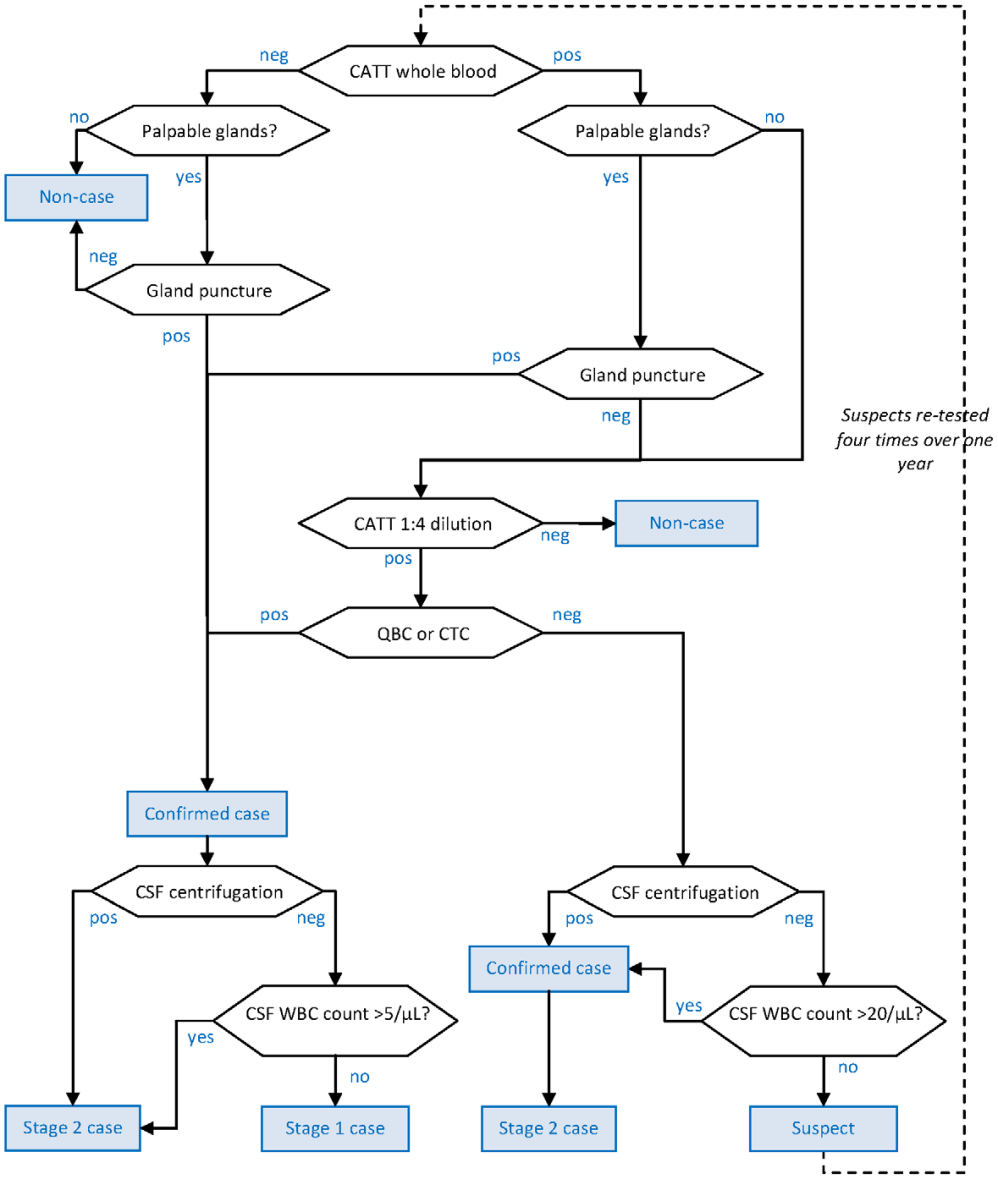
Diagnostic algorithm used in the Kiri, Southern Sudan programme (beginning of programme). Hexagonal boxes indicate tests. Square, blue-shaded boxes indicate points at which a decision on the patient is reached.

**Figure 3 pntd-0001233-g003:**
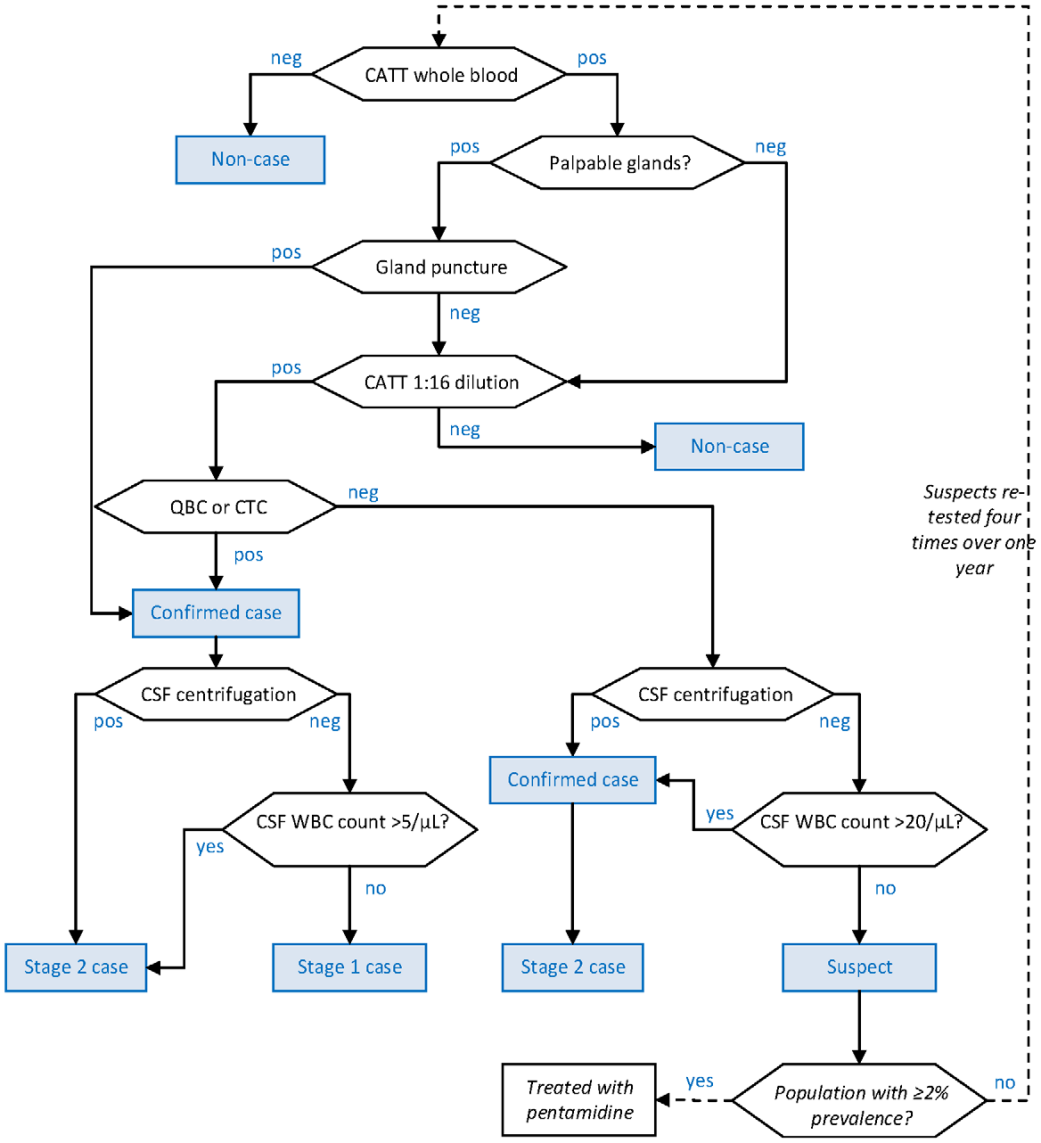
Diagnostic algorithm used in the Kiri, Southern Sudan programme (end of programme). Hexagonal boxes indicate tests. Square, blue-shaded boxes indicate points at which a decision on the patient is reached.

**Figure 4 pntd-0001233-g004:**
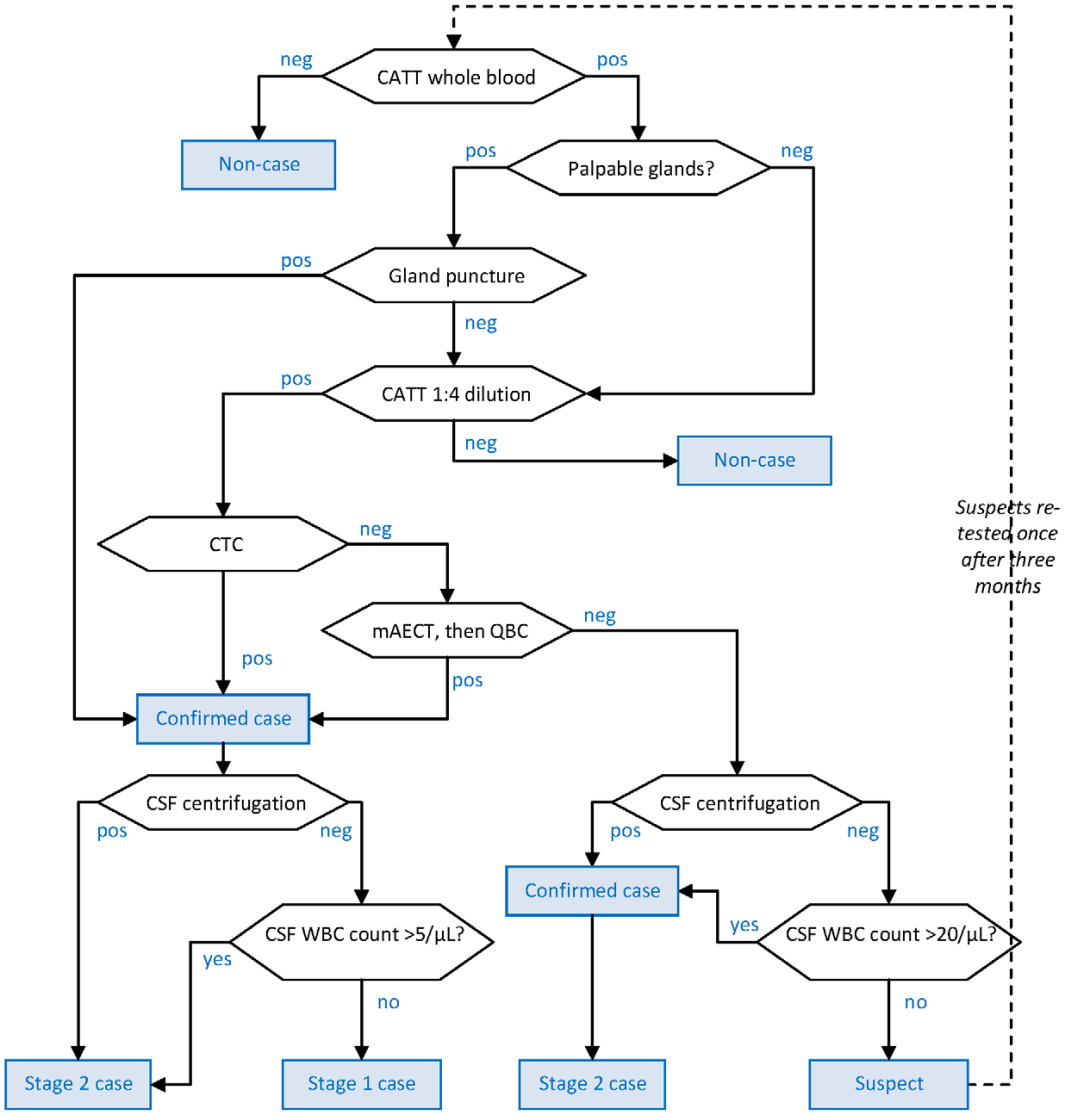
Diagnostic algorithm used by Adjumani programme, Uganda. Hexagonal boxes indicate tests. Square, blue-shaded boxes indicate points at which a decision on the patient is reached.

**Figure 5:**
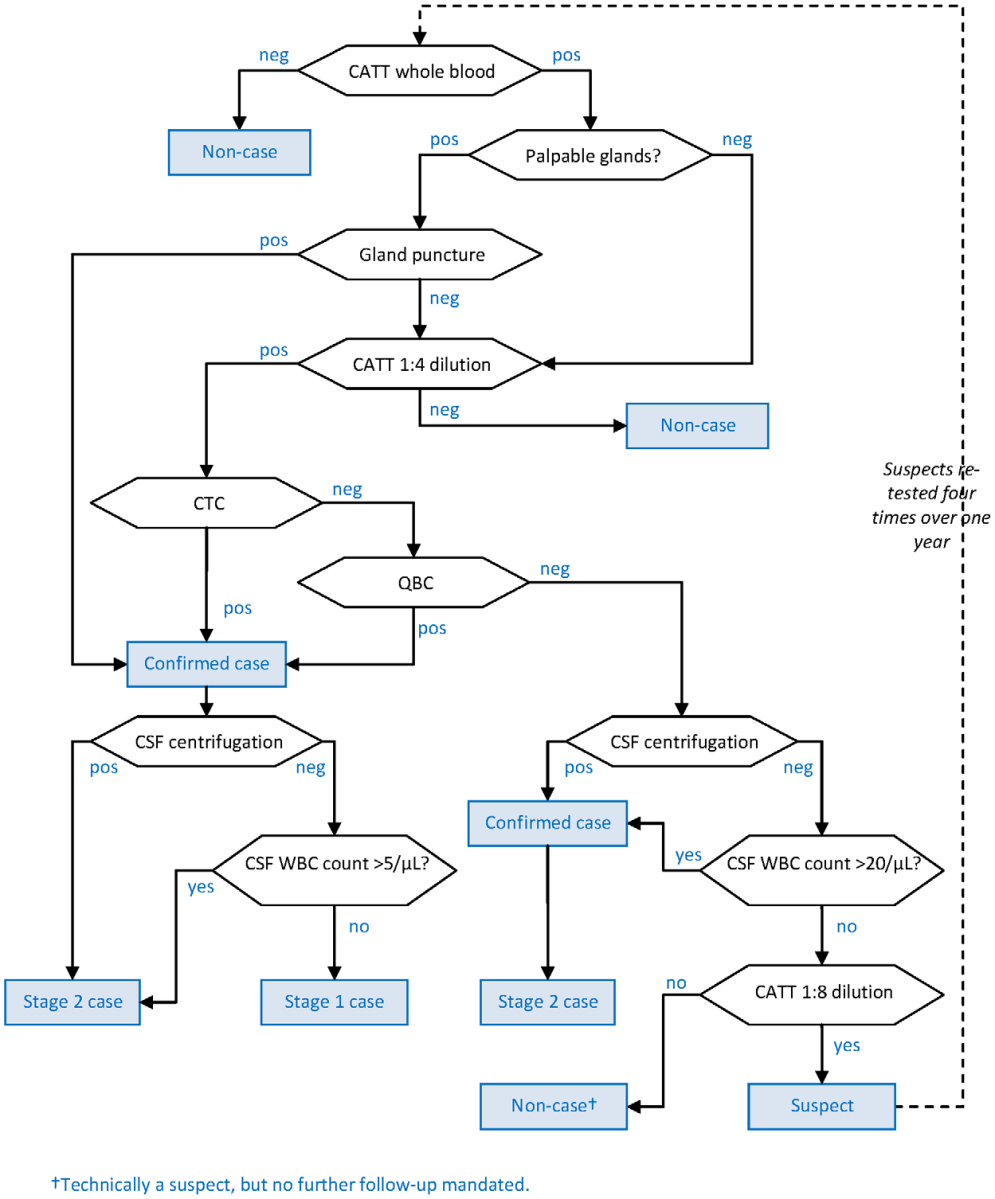
Diagnostic algorithm used by Arua-Yumbe programme, Uganda. Hexagonal boxes indicate tests. Square, blue-shaded boxes indicate points at which a decision on the patient is reached.

As initial screening tests, all algorithms used the CATT-wb, and the Congo and Sudan algorithms also used systematic gland palpation among CATT-wb negatives. Parasitology (performed on the field during active screening) included microscopic examination of aspirate from punctured palpable cervical glands (GP) [9], done in all algorithms, complemented by capillary tube centrifugation (CTC or the Woo test [10]; theoretical detection limit 100 parasites/mL, reported limit 500–600/mL) or the Quantitative Buffy Coat (QBC; 15/mL, 15–300/mL) technique [11] in Southern Sudan, and the mini anion exchange centrifugation technique (mAECT; 5/mL, 15–100/mL) [12] or QBC in Uganda. Furthermore, the Southern Sudan algorithms used the QBC as the parasitological test during passive screening (testing of patients spontaneously presenting to a HAT treatment centre), and the CTC during active screening.

All programmes initially did systematic follow-up of serological suspects, but this was eventually stopped in Congo and Kiri due to low follow-up rates and high workload; in Kiri, this strategy was replaced with systematic treatment of suspects positive at CATT dilution ≥1∶16, later restricted to villages with observed prevalence ≥2%. The Congo algorithm treated CATT≥1∶8 positive but T− individuals as cases regardless of CSF WBC density.

Staging of HAT in T+ (and CATT≥1∶8 positive in Congo) individuals was done at the fixed treatment centre by lumbar puncture and double centrifugation of the CSF (CSF-DC). If CSF-DC revealed no parasites, staging was based on WBC density thresholds. These thresholds were either >5 or >10/µL as per country guidelines [13].

With the exception of Congo, all algorithms performed LP in T− but CATT dilution (≥1∶4 or ≥1∶16) positive individuals for simultaneous confirmation and staging. For these patients, the WBC density threshold was increased to >20/µL; furthermore, those not meeting stage 2 criteria were not automatically considered stage 1 cases, but rather suspects, creating a differential in sensitivity according to whether the case was stage 1 or stage 2.

Differences among algorithms reflect adherence to national HAT guidelines (for example, in Congo the WBC threshold was higher); the availability on the market of certain parasitological tests at different times (for example, the mAECT is a more recent development and interruptions in the production line have occurred); different operational strategies (in Congo MSF aimed to cover a large, sparse territory with single active screening visits with the overriding objective of maximum coverage and thus sensitivity); and, to some extent, decisions by individual programme coordinators or MSF sections (in the past decade, an inter-sectional working group has worked toward greater standardisation).

### Literature review of the accuracy of individual tests

Medline PubMed searches were conducted with the MeSH terms “Trypanosomiasis, African/diagnosis”, and with combinations of [“trypanosomiasis”/“trypanosomosis”/“trypanosome”/“sleeping sickness”] and [“screening”/“confirmation”/“diagnosis”/“stage”/“staging”/“diagnostic”/“card agglutination test”/“CATT”/“gland”/“woo”/“capillary tube centrifugation”/“mini-anion exchange”/“buffy coat”/“cerebrospinal fluid”/“lumbar puncture”/“white blood cell”/“leucocyte”/“polymerase chain reaction”/“IgM”]. The bibliographic trail of each paper was followed to its exhaustion where appropriate, and several reviews [1], [14], [15] were consulted. The search was limited to the period from January 1970 to June 2007.

Studies were included in the review only if they had tested the accuracy of *T. brucei gambiense* diagnosis among untreated cases, and if they featured an acceptable diagnostic gold standard, defined as follows: (i) for screening and confirmation tests, testing with GP or CTC *and* at least one of the following: QBC, mAECT, enzyme linked immunosorbent assay (ELISA), Kit for In Vitro Identification (KIVI), or animal inoculation; (ii) for the specificity of the CATT-wb, testing of individuals not living in HAT endemic areas; (iii) for staging tests, testing of CSF, among T+ cases only, with polymerase chain reaction (PCR), in vitro culture, or immunological markers of infection including raised IgM levels [16].

Studies that were not designed for testing validity, but contained sufficient data for accuracy estimation, were included. In some studies, we considered the experimental test used by investigators as the gold standard, and vice versa: in these cases, we inverted the two and re-calculated accuracy. The accuracy of CATT dilutions was only evaluated from studies among CATT-wb positives, since the algorithms only performed such dilutions after the CATT-wb screening, i.e. the parameter of interest was *relative* accuracy compared to the CATT-wb. Reports of CATT accuracy from foci where parasites frequently lack the LiTat1.3 gene [1] (Nigeria, Cameroon) were excluded.

Details on studies meeting inclusion criteria are provided in Text S1, and the amount of information available for each diagnostic test is summarised in Table 1. An additional nine studies were excluded from either the sensitivity or specificity reviews because the gold standard was inadequate [17], [18], [19], [20], [21], [22] or the study design did not allow for diagnostic accuracy estimation [23], [24], [25]. One study of staging accuracy [26] was excluded because the IgM threshold used was deemed too high.

**Table 1:** Number of reports of sensitivity, specificity and staging accuracy contained in studies included in the review, by diagnostic test.

Diagnostic test	Sensitivity reports		Specificity reports	
	Number	References	Number	References
CATT-wb	8	[45], [46], [47], [48], [49], [50], [51], [52]	11 (8 used only in worst-case scenario)	[45], [53] ([45], [46], [47], [48], [49], [51], [52], [54])
CATT dilution 1∶2†	3	[4], [54], [55]	2	[54], [55]
CATT dilution 1∶4†	5	[4], [48], [54], [55], [56]	2	[54], [55]
CATT dilution 1∶5†	2	[34], [51]	0	
CATT dilution 1∶8†	4	[4], [54], [55], [56]	2	[54], [55]
CATT dilution 1∶10†	2	[34], [51]	0	
CATT dilution 1∶16†	4	[4], [54], [55], [56]	2	[54], [55]
CATT dilution 1∶20†	2	[34], [51]	0	
CATT dilution 1∶32†	4	[4], [54], [55], [56]	2	[54], [55]
CATT dilution 1∶40†	2	[34], [51]	0	
CATT dilution 1∶64†	3	[4], [54], [56]	1	[54]
CATT dilution 1∶80†	2	[34], [51]	0	
CATT dilution 1∶128†	1	[56]	0	
CATT dilution 1∶160†	2	[34], [51]	0	
CATT dilution 1∶320†	1	[51]	0	
GP	4	[34], [55], [57], [58]	0	
CTC	4	[34], [55], [58], [59]	1	[60]
mAECT	3	[34], [55], [58]	0	
QBC	1	[58]	0	
CSF-DC (if case is in stage 1 ‡)	5 (1 used only in worst-case scenario)	[34], [60], [61], [62] ([16])	5 (1 used only in worst-case scenario)	[34], [60], [61], [62] ([16])
CSF-DC (if case is in stage 2 ‡)	5	[16], [34], [60], [61], [62]		
WBC density >20/µL (if case is in stage 1 ‡)	4	[16], [61], [62], [63]	4	[16], [61], [62], [63]
WBC density >20/µL (if case is in stage 2 ‡)	4	[16], [61], [62], [63]		

	Number of stage 1 accuracy reports		Number of stage 2 accuracy reports	
	Number	References	Number	References
CSF-DC	5 (1 used only in worst-case scenario)	[34], [60], [61], [62] ([16])	5	[16], [34], [60], [61], [62]
WBC density >5/µL	4	[60], [61], [62], [63]	5	[25], [60], [61], [62], [63]
WBC density >10/µL	2	[61], [63]	2	[61], [63]
WBC density >20/µL	4	[16], [61], [62], [63]	4	[16], [61], [62], [63]

**†:** Among CATT-wb positives only.

**‡:** Stage 1 or 2 as defined according to the gold standard adopted for this study (see Methods).

### Probability distributions of test accuracy

Individual estimates of test accuracy were combined into probability distributions for further modelling. Distributions for the accuracy of successive CATT dilutions were constructed by fitting polynomial functions to plots of available sensitivity or specificity point estimates versus the natural logarithm of the dilution, with observations weighted proportionately to each study's sample size (Figure S1a, Figure S1b in Text S1). The fitted values and their 95% confidence intervals at each dilution of interest were used to construct binomial distributions.

**Figure 6:**
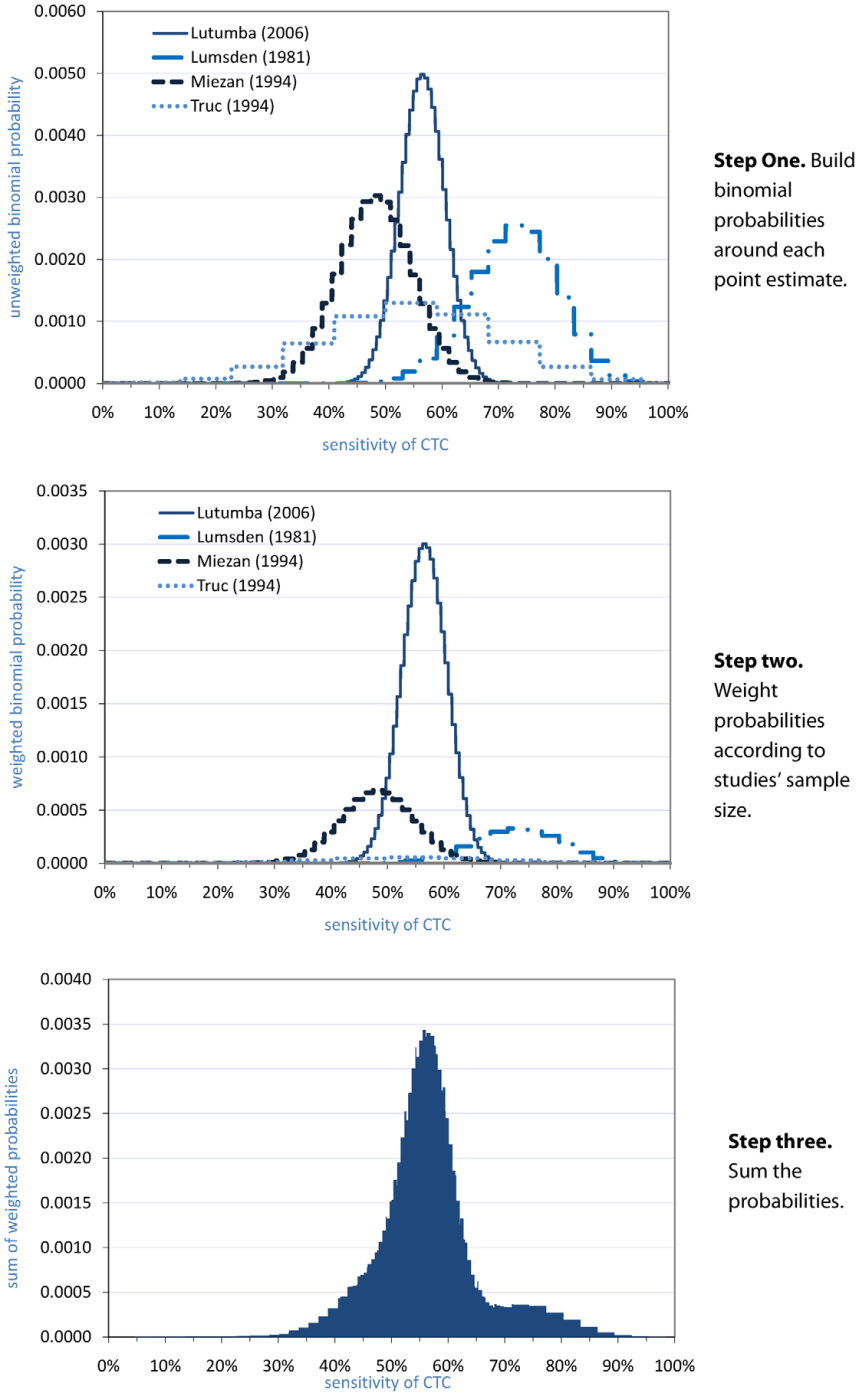
Steps to build a probability distribution of CTC test sensitivity. Each report is denoted by the name of the first author and the year of publication. In step three, the final probability distribution is then normalised to unity (i.e. the total probability = 1).

Probability distributions for other tests were constructed as follows. First, exact binomial probability distributions were built around the point estimate of each study. Second, each study's distribution was weighted proportionately to the study's sample size. Third, the individual study distributions were summed, and the resulting distribution was scaled so that the area under the curve totalled unity. An illustration is provided for the CTC (Figure 6).

For the QBC, there was only one published estimate of sensitivity, from a small study (n = 11). The technique is reported to have similar sensitivity to the mAECT [12], [20], which is plausible given their comparable detection limits: therefore, the same distribution was used for the QBC as for the mAECT.

Finally, the specificity of parasitological tests for confirmation was fixed at 100%: the presence of trypanosomes is unequivocal, and trained microscopists should ordinarily not report false positives.

### Alternative worst-case scenario

For the purpose of planning for long-term transmission control, it might be useful to consider minimum requirements to guarantee success even if conditions in reality are less favourable than published evidence suggests. Accordingly, more conservative accuracy estimates were obtained by applying a set of worst-case scenario assumptions (Table 2). These assumptions sought to account for the fact that even the most sensitive tests (QBC, mAECT) are likely to miss low parasitaemias (<5–15 trypanosomes/mL). Studies of T- suspects, based on PCR assays for *T. brucei* s.l. [27] featuring 100% specificity in controls from non-endemic regions [28], [29], [30], [31], have reported 22% positivity in Cameroon [30]; 19–37% in the Ivory Coast [29]; and 15% in Equatorial Guinea and Angola [32].

**Table 2:** Assumptions made in the worst-case scenario analysis.

Parameter	Rationale	Adjustment to baseline scenario
Sensitivity of CATT-wb	PCR evidence suggests some CATT-wb negative, T− individuals may in fact be infected (see Discussion: Plausibility of worst-case scenario assumptions)	1–5% lower than the baseline scenario (uniform distribution), based on range reported in the literature (see Discussion)
Sensitivity of parasitological tests performed after a first negative parasitological test (e.g. mAECT after negative CTC)	Average parasitaemia among cases not detected by the first test is probably lower: a greater proportion of those tested by the second test has parasitaemia below the test's detection limit	20–50% lower than the baseline scenario (uniform distribution); as no evidence was found, this range is assumed to be plausible
Sensitivity of algorithm among CATT-wb positive cases (i.e. of confirmation step)	PCR evidence suggests some infections are below the detection limit of parasitological tests (see Methods: Alternative worst-case scenario)	10–20% lower than the baseline scenario (uniform distribution), based on range of PCR positivity among T− suspects reported in the literature (see Methods)
Specificity of CATT-wb	Results from non-HAT exposed populations may be unrepresentative (e.g. HAT-exposed populations may also have higher prevalence of parasitic infections, such as *P. falciparum*, that may cross-react with the CATT [56])	Re-constructed probability distribution by including reports from apparently HAT-negative controls in HAT-endemic sites
Specificity of GP, CTC, mAECT, QBC, CSF-DC	Rare false positives could occur due to microscopic artefacts, e.g. microfilaria, or clerical mistakes	99.5–100.0% of the baseline scenario (uniform distribution); as no evidence was found, this range is assumed to be plausible
Staging accuracy of CSF-DC for stage 2	One study [16] reported much lower accuracy based on a gold standard consisting of various markers of neuro-inflammation including intrathecal IgM	Specificity from study in question (73.3%) adopted instead of those used in the baseline scenario
Sensitivity of CSF-DC for confirmation, if case is in stage 1	False CSF-DC positives would lead to confirmation of a patient as a case, even if the patient is in fact in stage 1	Sensitivity of 26.7% ( = 100%−73.3%) adopted, based on the above study

### Probabilistic model

R software was used to program the different algorithms into a sequence of conditional probabilities, so as to calculate sensitivity, specificity, and staging accuracy (defined as the probability of being correctly classified into either stage) of the algorithm as a whole, given any values of accuracy for individual tests. Equations for the accuracy estimation of each algorithm are provided in Text S1.

Because some algorithms used CSF-DC and WBC count for confirmation as well as staging, sensitivities vary according to whether the true positive case is in stage 1 or stage 2, and were thus computed separately. Furthermore, scenarios with and without follow-up of serological suspects were evaluated, i.e. assuming none or all such cases are re-tested according to the stipulated schedule (in practice, the follow-up rate varies by site [33]).

The sensitivity and specificity of any given test for the baseline scenario were specified by the probability distributions constructed above, summarised in Table 3. The model was run 10 000 times for each algorithm and for both the baseline and worst-case (incorporating the adjustments in Table 2) scenarios. During each run, a random value was sampled from each input probability distribution. Median sensitivity, specificity and staging accuracy were then computed based on the output distribution of results from the 10 000 runs, along with their 95% percentile interval (i.e. the interval comprising 95% of the run results).

**Table 3:** Input parameter values for baseline scenario.

Screening or confirmation test	Mean* or median sensitivity % (95% interval)	Mean* or median specificity % (95% interval)
CATT-wb	91.2 (78.1–99.8)	97.4 (93.8–99.2)
CATT 1∶4 dilution†	97.7* (92.8–100.0)	39.2* (29.6–48.8)
CATT 1∶8 dilution†	85.1* (81.0–89.2)	63.6* (55.2–72.0)
CATT 1∶16 dilution†	59.5* (55.2–63.7)	81.8* (72.0–91.6)
GP‡	58.5 (43.1–77.0)	100.0
CTC	56.0 (38.9–79.9)	100.0
mAECT	76.9 (68.8–92.1)	100.0
QBC	76.9 (68.8–92.1)	100.0
CSF-DC if case is in stage 1	2.4 (0.0–13.6) ¶	100.0
CSF-DC if case is in stage 2	68.8 (50.6–85.2)	
WBC density >20/µL if case is in stage 1	3.6 (0.1–17.8) ¶	96.2 (82.0–99.7)
WBC density >20/µL if case is in stage 2	67.3 (32.3–75.5)	

Staging test	Median accuracy of stage 1 classification % (95% interval)	Median accuracy of stage 2 classification % (95% interval)
CSF-DC	97.4 (86.2–99.8) ¶	68.8 (50.6–85.2)
WBC density >5/µL	69.8 (45.1–87.8)	74.4 (46.3–89.8)
WBC density >10/µL	90.3 (58.4–98.4)	65.6 (39.1–80.7)
WBC density >20/µL	96.2 (82.0–99.7) ¶	67.3 (32.3–75.5)

Prevalence of palpable cervical glands	Prevalence in % (range†)	Distribution	Notes
Among true positives	50.0–100.0	uniform	[1], [64]
Among true negatives	0.0–10.0	uniform	Based on prevalence of glands among CATT-wb negative non-cases in HAT foci under control in DRC [65], [66]

**†:** Among CATT-wb positives.

**‡:** Among persons with palpable glands.

**¶:** Accuracy is <100% in these cases because of a study reporting a small percentage of false positives, based on the gold standard stage definition adopted in this study (see Methods). If the patient is in fact a true case and his or her infection is not confirmed by any other test, these false positives would make a very small, serendipitous sensitivity contribution. Conversely, they would result in a less than perfect staging accuracy.

The resulting negative and positive predictive values (NPV, PPV) were also calculated assuming 0.1%, 1% or 10% infection prevalence. The ratio of non-cases needlessly treated to true cases treated (over-treatment ratio) was also calculated for each algorithm and prevalence scenario, assuming a stage 1 to stage 2 ratio of two among prevalent infections detected actively in never-before screened communities, consistent with empirical observations in most MSF projects (Francesco Checchi, unpublished observations). However, this assumption is of negligible importance: the converse (a ratio of 0.5) would result in nearly identical estimates (data not shown), since differences in sensitivity between stage 1 and stage 2 are small and of limited influence given that HAT is a low-prevalence infection (PPV and NPV are largely determined by specificity).

## Results

### Sensitivity, specificity and staging accuracy

Accuracy estimates for the baseline scenario are shown in Table 4. Sensitivity including suspect follow-up was highest in Congo, and considerably lower than elsewhere for the new Kiri algorithm, which screened out cases negative at a high CATT dilution (<1∶16). Specificity was 99.9% or 100% everywhere with the exception of Congo (99.1%).

**Table 4:** Estimated sensitivity, specificity and accuracy of staging of HAT diagnostic algorithms (baseline scenario).

Accuracy indicator	Gamboma, Mossaka, Nkayi, Republic of Congo	Kiri, Sudan (old algorithm)	Kiri, Sudan (new algorithm)	Adjumani, Uganda	Arua/Yumbe, Uganda
**Screening and confirmation of infection, assuming perfect follow-up of suspects** †					
Sensitivity if case is stage 1 (%)	95.2 (87.6–99.8)	QBC: 93.8 (84.6–99.3)CTC: 93.8 (84.4–99.2)	QBC: 70.3 (57.6–83.2)CTC: 69.9 (56.5–83.2)	89.0 (75.5–98.8)	89.3 (76.0–98.5)
Sensitivity if case is stage 2 (%)	95.2 (87.6–99.8)	QBC: 93.8 (84.7–99.3)CTC: 93.9 (84.9–99.3)	QBC: 70.4 (57.9–83.2)CTC: 70.3 (57.8–83.2)	89.6 (76.5–99.1)	89.7 (76.5–99.0)
Specificity (%)	99.1 (97.7–99.7)	QBC: 99.9 (99.6–100.0)CTC: 99.9 (99.6–100.0)	QBC: 100.0 (99.9–100.0)CTC: 100.0 (99.9–100.0)	99.9 (99.6–100.0)	99.9 (99.6–100.0)
**Screening and confirmation of infection, assuming no follow-up of suspects**					
Sensitivity if case is stage 1 (%)	92.1 (84.2–97.1)	QBC: 83.9 (72.7–93.2)CTC: 73.8 (60.1–87.3)	QBC: 64.1 (51.1–79.2)CTC: 58.0 (43.5–75.0)	85.3 (72.3–96.1)	85.6 (72.5–96.0)
Sensitivity if case is stage 2 (%)	92.1 (84.2–97.1)	QBC: 92.0 (82.5–97.8)CTC: 90.0 (80.0–96.5)	QBC: 69.1 (56.7–82.3)CTC: 67.9 (55.2 (81.4)	88.8 (75.9–98.3)	89.1 (75.8–98.4)
Specificity (%)	99.1 (98.0–99.7)	QBC: 99.9 (99.7–100.0)CTC: 99.9 (99.7–100.0)	QBC: 100.0 (99.9–100.0)CTC: 100.0 (99.9–100.0)	99.9 (99.7–100.0)	99.9 (99.7–100.0)
**Accuracy of staging**					
Probability of being correctly classified as stage 1 (%)	87.0 (61.1–95.9)	QBC: 66.5 (43.9–84.3)CTC: 65.9 (43.2–83.3)	QBC: 66.6 (44.1–84.7)CTC: 66.0 (43.6–83.3)	66.9 (43.9–85.0)	67.0 (44.1–84.9)
Probability of being correctly classified as stage 2 (%)	89.0 (79.0–94.8)	QBC: 92.9 (82.2–97.7)CTC: 93.7 (84.2–98.0)	QBC: 92.3 (81.9–97.7)CTC: 93.4 (83.6–97.9)	92.4 (81.2–97.6)	92.4 (81.6–97.6)

**†:** Four follow-up visits at three month intervals in all projects except for Adjumani (one visit at three months only). Under the new Kiri algorithm, suspect follow-up only occurred if the village has an observed prevalence >2%.

Values in parentheses are 95% percentile intervals.

The theoretical sensitivity gain from suspect follow-up was considerable: about 3–4% everywhere, but 10–20% in Kiri, where T−, CATT dilution ≥1∶4 positives were followed up. There was no appreciable specificity cost from suspect follow-up. Algorithms were predicted to misclassify about one in ten of the stage 2 cases as stage 1; conversely, about one third of stage 1s were treated as stage 2, with the exception of Congo, where the higher WBC threshold (>10/µL) resulted in a small increase in stage 2 misclassification, but only 13% stage 1 misclassification (note however the wide percentile intervals).

In the worst-case scenario (Table 5), sensitivity was 10–15% lower everywhere except for Congo (where conservative assumptions mostly did not affect the CATT≥1∶8 dilution test), and around 50% for the new Kiri algorithm. Specificity decreased below 99.8% except for the new Kiri algorithm. Stage misclassification affected more than half of stage 1 cases.

**Table 5:** Estimated sensitivity, specificity and accuracy of staging of HAT diagnostic algorithms (worst-case scenario).

Accuracy indicator	Gamboma, Mossaka, Nkayi, Republic of Congo	Kiri, Sudan (old algorithm)	Kiri, Sudan (new algorithm)	Adjumani, Uganda	Arua/Yumbe, Uganda
**Screening and confirmation of infection, assuming perfect follow-up of suspects** †					
Sensitivity if case is stage 1 (%)	91.7 (83.5–97.1)	QBC: 81.8 (76.6–89.8)CTC: 80.7 (70.5–88.9)	QBC: 59.6 (47.7–72.5)CTC: 58.7 (46.2–72.1)	74.3 (61.1–86.3)	75.4 (62.8–86.2)
Sensitivity if case is stage 2 (%)	91.7 (83.5–97.1)	QBC: 80.6 (70.4–88.7)CTC: 79.0 (69.0–87.6)	QBC: 59.0 (47.4–71.9)CTC: 58.1 (46.1–71.2)	75.6 (63.1–86.7)	76.1 (63.5–86.9)
Specificity (%)	97.8 (97.1–99.5)	QBC: 99.6 (99.0–99.9)CTC: 99.6 (99.0–99.9)	QBC: 99.9 (99.8–100.0)CTC: 99.9 (99.8–100.0)	99.7 (99.2–100.0)	99.7 (99.2–100.0)
**Screening and confirmation of infection, assuming no follow-up of suspects**					
Sensitivity if case is stage 1 (%)	85.2 (76.7–91.4)	QBC: 71.1 (60.0–81.7)CTC: 64.3 (52.3–77.0)	QBC: 53.3 (41.5–67.3)CTC: 49.2 (36.9–64.3)	67.8 (55.6–80.3)	69.5 (56.7–81.3)
Sensitivity if case is stage 2 (%)	85.2 (76.7–91.4)	QBC: 77.8 (67.4–86.6)CTC: 74.7 (64.1–84.3)	QBC: 57.4 (45.8–70.5)CTC: 55.6 (43.5–69.1)	73.7 (61.6–85.0)	74.6 (61.9–85.6)
Specificity (%)	97.8 (97.3–99.5)	QBC: 99.6 (99.1–99.9)CTC: 99.6 (99.1–99.9)	QBC: 99.9 (99.8–100.0)CTC: 99.9 (99.8–100.0)	99.7 (99.2–100.0)	99.7 (99.2–100.0)
**Accuracy of staging**					
Probability of being correctly classified as stage 1 (%)	63.6 (44.6–74.1)	QBC: 47.5 (30.5–62.4)CTC: 45.8 (29.4–60.4)	QBC: 47.8 (31.1–62.9)CTC: 46.6 (30.1–61.3)	47.5 (30.7–62.3)	48.0 (31.0–63.0)
Probability of being correctly classified as stage 2 (%)	89.2 (78.9–94.8)	QBC: 93.1 (83.0–97.8)CTC: 93.7 (84.4–98.0)	QBC: 93.0 (82.7–97.8)CTC: 93.5 (83.6–98.0)	92.9 (82.8–97.8)	92.8 (82.3–97.8)

**†:** Four follow-up visits at three month intervals in all projects except for Adjumani (one visit at three months only). Under the new Kiri algorithm, suspect follow-up only occurred if the village has an observed prevalence >2%.

Values in parentheses are 95% percentile intervals.

Overall, the Congo and new Kiri algorithms offered opposite extreme characteristics: the former guaranteed very high sensitivity but had low specificity; the latter was highly specific even under worst-case scenario assumptions, but had low sensitivity.

### Predictive values and over-treatment ratios

NPV was uniformly high (Table 6). Because of low specificity, the predicted PPV of the Congo algorithm was also low at most plausible prevalence levels (<50% for any prevalence <1%), resulting in a high over-treatment ratio. Because PPV is extremely sensitive to minimal changes in specificity, predicted PPVs with high specificity values should be interpreted with caution (e.g. in Uganda, median specificity was 99.94%, but was rounded to 99.9%, which results in a 20% decrease in PPV at prevalence 0.1%). Only the new Kiri algorithm achieved perfect PPV at any prevalence (however, the resultant elimination of over-treatment was counterbalanced by a policy of treating serological suspects with pentamidine in high-prevalence villages).

**Table 6:** Predictive values and over-treatment ratio for each algorithm, at three different prevalence levels.

Accuracy indicator	Infection prevalence (%)†	Republic of Congo‡	Kiri, Southern Sudan (old algorithm) with QBC (with CTC¶)	Kiri, Southern Sudan (new algorithm‡) with QBC (with CTC¶)	Adjumani, Uganda	Arua-Yumbe, Uganda
**Baseline scenario**						
Positive predictive value (%)	0.1	9.3	48.4 (48.4)	100.0 (100.0)	47.2	47.3
	1.0	50.8	90.5 (90.5)	100.0 (100.0)	90.0	90.0
	10.0	91.9	99.1 (99.1)	100.0 (100.0)	99.0	99.0
Negative predictive value (%)	0.1	100.0	100.0 (100.0)	100.0 (100.0)	100.0	100.0
	1.0	99.9	99.9 (99.9)	99.7 (99.6)	99.9	99.9
	10.0	99.1	99.3 (99.3)	96.4 (96.1)	98.8	98.9
Ratio of false to true cases treated	0.1	9.8	1.1 (1.1)	0.0 (0.0)	1.1	1.1
	1.0	1.0	0.1 (0.1)	0.0 (0.0)	0.1	0.1
	10.0	0.1	0.01 (0.01)	0.0 (0.0)	0.01	0.01
**Worst-case scenario**						
Positive predictive value (%)	0.1	3.7	16.9 (16.7)	37.2 (36.9)	20.0	20.2
	1.0	28.1	67.2 (66.9)	85.7 (85.5)	71.6	71.8
	10.0	81.1	95.8 (95.7)	98.5 (98.5)	96.5	96.6
Negative predictive value (%)	0.1	100.0	100.0 (100.0)	100.0 (100.0)	100.0	100.0
	1.0	99.9	99.8 (99.8)	99.6 (99.6)	99.8	99.8
	10.0	98.4	98.0 (97.8)	95.7 (95.6)	97.3	97.4
Ratio of false to true cases treated	0.1	25.8	4.9 (5.0)	1.7 (1.7)	4.0	4.0
	1.0	2.6	0.5 (0.5)	0.2 (0.2)	0.4	0.4
	10.0	0.2	0.04 (0.05)	0.02 (0.02)	0.04	0.04

**†:** Assumed stage 1 to stage 2 ratio of two. Note that a ratio of 0.5 would result in almost identical estimates (data not shown), since the differences in sensitivity between stage 1 and 2 are small and have limited influence on the PPV and NPV calculations given the low prevalence of HAT true positives.

**‡:** Assuming sensitivity and specificity without suspect follow-up, as done in practice.

**¶:** CTC values in parentheses.

## Discussion

### Interpretation of findings

This study suggests that diagnostic algorithms previously used by MSF had a sensitivity of 85–90% in a baseline scenario analysis, except for an algorithm in Southern Sudan in which only individuals CATT≥1∶16 positive underwent blood and CSF parasitological exams. At least theoretically, and irrespective of its efficiency and cost-effectiveness, the follow-up of serological suspects does yield an appreciable increase in sensitivity; however, this benefit may largely be negated in the field because of low suspect follow-up rates (suspect follow-up is costly as it often requires active patient tracing). Among other studies of HAT diagnostic algorithms (all starting with CATT-wb positivity), Miezan et al. [34] found sensitivities of 94.8%, 98.3% and 91.4% for the [GP+CTC+CSF-DC], [GP+mAECT+CSF-DC] and [GP+mAECT] combinations, respectively; Robays et al. projected sensitivity 76.6% for the mAECT [35]; Lutumba et al. estimated a sensitivity of 86% for the [GP+CTC] combination [36].

All algorithms also appeared to have an acceptable PPV except for Congo's, where serological diagnosis probably resulted in a high frequency of stage 1 false positives (see below). Furthermore, reliance on the conventional HAT staging approach (parasitology and WBC threshold of >5 leucocytes/µL) may have captured the vast majority of stage 2 cases but misclassified about one third of stage 1 cases as stage 2: this harm-benefit ratio is nonetheless likely to be favourable, since the risk of death from undetected stage 2 HAT is probably 100% [37], while the risk of death due to stage 2 drug toxicity among stage 1 cases misclassified as stage 2 is less than 5%, and <2% wherever eflornithine-nifurtimox has replaced melarsoprol as first-line treatment. Misclassification of stage 2 cases could partly be avoided by introducing some clinical criteria in the algorithm (e.g. patients with typical signs and symptoms of stage 2, and who are classified as stage 1, should be retested or treated empirically).

Our findings refer to the relatively favourable conditions of HAT diagnosis provided for by a well-resourced non-governmental organisation with access to the best available technology, ability to train and supervise staff and considerable field logistics. Many HAT programmes, particularly those implemented by national control programmes after humanitarian agencies and other donors discontinue support, do not dispose of such resources, and must use simpler algorithms, sometimes relying on blood smears and cervical node microscopy alone for parasitological testing in remote active screening campaigns. Such simple algorithms are likely to feature a much lower accuracy than those we have evaluated here: national programmes should receive continued technical and material support in order to offer adequate HAT diagnosis.

### Plausibility of worst-case scenario assumptions

While worst-case scenario estimates may be implausibly low, the question of whether current tests miss a larger proportion of cases than currently thought, as suggested by PCR data, should be explored further. While in non-endemic areas PCR appears extremely specific, among CATT-wb negatives in endemic areas PCR positives do occur: 4/73 (5.5%) in Ivory Coast [29], 3/222 (1.4%) in Cameroon [30], and 1/36 (2.8%) in Equatorial Guinea and Angola [32]. These observations could be explained as (i) false PCR positives due to cross-reactivity with other antigens, including DNA from non-gambiense *T. brucei* s.l. transiently infecting the host; or (ii) true *T. b. gambiense* infections undetectable by other tests due to low parasite density.

The former explanation is supported by the finding that a study in an Ivory Coast focus employing a PCR assay specific for *T. b. gambiense* yielded no PCR positives [31], while all studies with high PCR positivity relied on non-gambiense specific assays. However, the Ivory Coast assay used had a detection limit comparable to the mAECT, and may have failed to detect cases of low parasitaemia (by contrast, the non-gambiense specific Cameroon assay developed by Penchenier et al. [30] has a reported limit of 1/mL).

The latter explanation requires the existence of infections that maintain extremely scanty parasitaemia and are not or only mildly pathogenic [37].

Better evidence should come from the development of *T.brucei gambiense* specific molecular assays that also have a detection limit appreciably lower than parasitology, and their application to long-term follow-up of T− serological suspects [38]. Estimating the true sensitivity of tests would require knowledge of the typical distribution of parasitaemias in human hosts, but this is difficult to measure precisely because of the detection limit of current methods (presumably, if a large database of known parasite densities were assembled, the resulting distribution could be treated as truncated, and extrapolated below the minimum detection limit). Data on cattle are available, but may not apply to humans due to differences in host-parasite interactions.

In the mean time, we suggest that worst-case assumptions be used for determining requirements of programmes aiming for long-term control or local elimination.

### Implications for field diagnosis

Specificity is key to maximising PPV. Very low HAT infection prevalence (e.g. <0.2%) is common in many communities screened actively, implying poor PPV, considerable over-treatment, and inflated prevalence estimates for even the most specific algorithms considered here. However, in many programmes the majority of cases are detected passively. The prevalence of infection among individuals spontaneously presenting to the fixed HAT centre is higher, and was above 2% in all MSF programmes where these algorithms were used (Table 7). These observed prevalence figures suggest that PPV is generally high during passive screening (>95% everywhere except Congo).

**Table 7:** Prevalence of stage 1 and 2 HAT infection among persons screened passively in five MSF programmes.

Programme	Stage 1	Stage 2	Total cases†	Total screened passively†	Prevalence of stage 1	Prevalence of stage 2	Overall prevalence
**Gamboma**	19	37	56	2028	0.9%	1.8%	2.8%
**Nkayi**	107	162	269	10 552	1.0%	1.5%	2.6%
**Kiri**	792	1269	2061	43 562	1.8%	2.9%	4.7%
**Adjumani**	660	1732	2392	22 175	3.0%	7.8%	10.78%
**Arua-Yumbe**	327	1539	1866	39 465	0.8%	3.9%	4.7%
**Total**	**1905**	**4739**	**6644**	**117 782**	**1.6%**	**4.0%**	**5.6%**

**†:** Data on numbers screened passively are incomplete: cases are tallied here only if the number of persons screened passively during the same month is known.

Assuming reasonable laboratory quality, all parasitological tests are likely to be 100% specific, and reliance on these alone for confirmation should guarantee perfect PPV. By contrast, this study suggests that use of a CATT 1∶8 dilution positive test as criterion for confirming infection, irrespective of parasitological results, entails a heavy specificity price. Field data appear to corroborate this finding. Among true cases, the proportion diagnosed via the CATT 1∶8 dilution (serologically) should in theory not depend on HAT stage (serological tests in blood are believed by some to be less sensitive in stage 2, but no published evidence for this was found). On the other hand, among false positives, most cases diagnosed serologically would be classified as stage 1, since during staging all would be negative for CSF-DC and most would have normal WBC density. A preponderance of stage 1 is thus indicative of considerable over-diagnosis. Within the three Congo sites, serological cases were 1559/2857 (54.6%) of naïve (previously untreated) cases, of which 1364/1559 (87.5%) were in stage 1, compared to 624/1298 (48.1%) of cases confirmed parasitologically. Furthermore, serological cases were 244/629 (38.8%) of cases detected passively, and 1244/2152 (57.8%) of cases detected actively. In a simple logistic regression model, both stage 1 classification and active screening were associated with serological diagnosis (odds ratios 7.45 [95%CI 6.13–9.05] and 1.35 [95%CI 1.10–1.66] respectively). Altogether, these observations suggest considerable over-diagnosis of HAT (nearly all classified as stage 1) in Congo. Inojosa et al. found a similarly low PPV of an algorithm based on the CATT 1∶8 dilution in Angola (13.2% with 0.07% prevalence) [22]. Diagnosis through CATT serology does improve sensitivity considerably; however, we suggest that its use be restricted to (i) passive screening and (ii) active screening in remote communities with suspected high prevalence where there is likely to be only one opportunity for screening, and where melarsoprol is not used as first-line therapy or the algorithm minimises misclassification of stage 1 cases. Furthermore, we recommend use of a 1∶16 dilution in lieu of 1∶8. Control programs that use algorithms with serological criteria aim to reduce transmission at the expense of over-treatment. However, the individuals diagnosed solely on serology should not be regarded as HAT cases for the calculation of prevalence, as this would result in an overestimation of disease burden and obscure prevalence changes over time. They should be clearly distinguished from genuine cases in programme reporting and surveillance.

The main reason for lack of sensitivity of the parasitological tests is likely to be low parasite density. As HAT parasitaemia is known to undulate on a daily basis, some laboratories perform repeat blood parasitological tests so as to increase chances of detecting parasites. Repeat tests could be a simple way to improve sensitivity. Better evidence on the typical period between peak and trough parasitaemia would be helpful to optimise the timing of blood sampling. Clearly, keeping suspects for days at the treatment centre in order to repeat tests would present serious acceptability challenges; however, a single overnight might be feasible, and, furthermore, the selection of suspects in whom to perform repeat tests might also be restricted to those displaying typical signs and symptoms of HAT.

These findings also have implications for burden estimation, since they introduce a need to adjust observed prevalence or incidence data for imperfect sensitivity, PPV below 100% due to low specificity (particularly for active screening data), and unequal stage 1 and stage 2 misclassification probabilities.

### Study limitations

The literature review revealed a dearth of quality studies of HAT test accuracy, with the exception of the CATT-wb. Many were imprecise (only two presented a sample size rationale) and featured less than optimal gold standards. The mAECT, used in a variety of programmes, appears to be supported by only one large study, and for the QBC only one study was found. This uncertainty may introduce information bias in the construction of accuracy distributions. More specifically, the adoption of specificity estimates for the CATT from populations from non-endemic areas may have led to overly optimistic estimates (this was partly addressed in the worst case scenario analysis).

Our method of constructing accuracy distributions attempts to use existing data with minimal assumptions about their parametric form. Arguably, meta-analysis could have been used instead, with distributions provided by the confidence intervals of the summary estimates from pooled studies. However, preliminary analysis showed evidence of heterogeneity in study estimates for several HAT tests: under these conditions, meta-analysis is discouraged. Furthermore, there is lack of consensus on appropriate methods for meta-analysis of diagnostic test studies [39], [40].

Bayesian approaches to diagnostic accuracy estimation [41], [42], which do not require a gold standard, could be a useful alternative to the method used here, and should also be explored.

More generally, this study's theoretical estimates overlook some practical realities of field work. For example, algorithms are sometimes not performed as indicated (e.g. gland palpation may be skipped due to heavy workload); some diagnostic decisions are taken on clinical grounds (though probably rarely), overriding laboratory results; and patient attrition is an issue (e.g. suspect follow-up rates are generally low). Thus, the algorithms' accuracy in routine conditions may be higher or lower than our estimates, the latter being more likely.

### Conclusions

Algorithms using non-parasitological diagnosis have lower specificity leading to varying degrees of overtreatment. Overestimation of disease burden could be avoided by excluding individuals diagnosed serologically from the case counts. Differences between active and passive screening should be considered. Ways to improve sensitivity include follow-up of serological suspects and repeat blood parasitological testing. This study highlights the urgent need to pursue research on new HAT diagnostics [43]. Improved tests should ideally replace most of the present algorithms, and be feasible in outpatient settings (e.g. as simple serological rapid tests), thus enabling integration of HAT services [44]. In the present scenario of falling prevalence, any new tests will need to be practically 100% specific. However, high sensitivity will remain necessary to maximise the chances of elimination. No single algorithm will be appropriate for all epidemiological settings: rather, our study demonstrates the value of estimating the accuracy of the algorithm as a whole, and could be replicated in a variety of prevalence scenarios, or integrated in a cost-effectiveness analysis that would help control programmes, particularly those working with limited resources, optimise the use of available diagnostics.

## Supporting Information

Text S1 Model equations and results of the diagnostic literature review.
(DOC)
