# Supplementary material for: Accuracy of Five Algorithms to Diagnose Gambiense Human African Trypanosomiasis
Source: PLoS Negl Trop Dis. 2011 Jul 5;5(7):e1233. doi: 10.1371/journal.pntd.0001233 (PMC3130008; doi:10.1371/journal.pntd.0001233)
Supplement: Text S1 — Model equations and results of the diagnostic literature review. (DOC) [file pntd.0001233.s001.doc]

**Accuracy of five algorithms to diagnose gambiense human African trypanosomiasis**

**Supporting Information S1**

**Equations for the probabilistic model**

Table S1a. Symbols used in the equations.

| **Symbol** |  | **Meaning** |
| --- | --- | --- |
| Σ |  | sensitivity of confirmation |
| Φ |  | specificity of confirmation |
| σ’ |  | sensitivity of confirmation, assuming no suspect follow-up |
| φ’ |  | specificity of confirmation, assuming no suspect follow-up |
| Ψ |  | accuracy of staging classification |
| s1 |  | subscript indicating accuracy if case is in stage 1 |
| s2 |  | subscript indicating accuracy if case is in stage 2 |
| g_TP_ |  | prevalence of cervical glands among true cases |
| g_FP_ |  | prevalence of cervical glands among true non-cases |
| SS_TP_ |  | serological suspects among true cases |
| SS_FP_ |  | serological suspects among true non-cases |
| N |  | number of theoretical follow-up visits if one is a suspect |
| L |  | left branch of algorithm |
| R |  | right branch of algorithm |

**Gamboma, Mossaka, Nkayi, Republic of Congo**

Sensitivity of confirmation (no suspect follow-up):

Sensitivity of confirmation (with suspect follow-up):

$SS_{TP}=[g_{TP}\sigma_{CATT-wb}(1-\sigma_{GP})+(1-g_{TP})\sigma_{CATT-wb}](1-\sigma_{CTC})(\sigma_{CATT1:4})(1-\sigma_{CATT1:8})$

$\sigma=\sigma'+SS_{TP}[1-(1-\sigma')^{n}]$, where n=4

Specificity of confirmation (no suspect follow-up):

Specificity of confirmation (with suspect follow-up):

$$SS_{FP}=[g_{FP}(1-\phi_{CATT-wb})\phi_{GP}+(1-g_{FP})(1-\phi_{CATT-wb})]\phi_{CTC}(1-\phi_{CATT1:4})\phi_{CATT1:8}$$

$$\phi=\phi'+SS_{FP}(1-\phi'^{n})]$$

Accuracy of stage 1 classification:

$$\psi_{s1}=\psi_{s1,LP-DC}\psi_{s1,WBC>10}$$

Accuracy of stage 2 classification:

$$\psi_{s2}=\psi_{s2,LP-DC}+(1-\psi_{s2,LP-DC})\psi_{s2,WBC>10}$$

**Kiri, Sudan (old algorithm)**

*[assuming QBC is used; otherwise, substitute QBC with CTC in all equations]*

Sensitivity of confirmation, if case is in stage 1 (no suspect follow-up):

*Left branch of algorithm:*

$$\sigma'_{s1}(L)=g_{TP}\sigma_{GP}+[(1-g_{TP})\sigma_{CATT-wb}+g_{TP}\sigma_{CATT-wb}(1-\sigma_{GP})]\sigma_{CATT1:4}\sigma_{QBC}$$

*Right branch of algorithm:*

*Overall:*

$$\sigma'_{s1}=\sigma'_{s1}(L)+\sigma'_{s1}(R)$$

Sensitivity of confirmation, if case is in stage 2 (no suspect follow-up):

*Left branch of algorithm:*

$$\sigma'_{s2}(L)=\sigma'_{s1}(L)$$

*Right branch of algorithm:*

*Overall:*

$$\sigma'_{s2}=\sigma'_{s2}(L)+\sigma'_{s2}(R)$$

Sensitivity of confirmation, if case is in stage 1 (with suspect follow-up):

$\begin{matrix} SS_{TP,s1}=[(1-g_{TP})\sigma_{CATT-wb}+g_{TP}\sigma_{CATT-wb}(1-\sigma_{GP})]\sigma_{CATT1:4}(1-\sigma_{QBC})(1-\sigma_{LP-DC,s1}) \\ (1-\sigma_{WBC>20,s1}) \end{matrix}$

$\sigma_{s1}=\sigma'_{s1}+SS_{TP,s1}[1-(1-\sigma'_{s1})^{n}]$, where n=4

Sensitivity of confirmation, if case is in stage 2 (with suspect follow-up):

$\begin{matrix} SS_{TP,s2}=[(1-g_{TP})\sigma_{CATT-wb}+g_{TP}\sigma_{CATT-wb}(1-\sigma_{GP})]\sigma_{CATT1:4}(1-\sigma_{QBC})(1-\sigma_{LP-DC,s2}) \\ (1-\sigma_{WBC>20,s2}) \end{matrix}$

$\sigma_{s2}=\sigma'_{s2}+SS_{TP,s2}[1-(1-\sigma'_{s2})^{n}]$, where n=4

Specificity of confirmation, if case is in stage 1 (no suspect follow-up):

*Left branch of algorithm:*

$$\phi'_{s1}(L)=1-g_{FP}(1-\phi_{GP})-[(1-g_{FP})(1-\phi_{CATT-wb})+g_{FP}\phi_{GP}](1-\phi_{CATT1:4})(1-\phi_{QBC})$$

*Right branch of algorithm:*

*Overall:*

$$\phi'_{s1}=\phi'_{s1}(L)+\phi'_{s1}(R)$$

Specificity of confirmation, if case is in stage 2 (no suspect follow-up):

*Left branch of algorithm:*

$$\phi'_{s2}(L)=\phi'_{s1}(L)$$

*Right branch of algorithm:*

*Overall:*

$$\phi'_{s2}=\phi'_{s2}(L)+\phi'_{s2}(R)$$

Specificity of confirmation, if case is in stage 1 (with suspect follow-up):

$SS_{FP,s1}=[(1-g_{FP})(1-\phi_{CATT-wb})+g_{FP}\phi_{GP}](1-\phi_{CATT1:4})\phi_{QBC}\phi_{LP-DC,s1}\phi_{WBC>20,s1}$

$\phi_{s1}=\phi'_{s1}+SS_{FP,s1}(1-\phi'_{s1})^{n}]$, where n=4

Specificity of confirmation, if case is in stage 2 (with suspect follow-up):

$SS_{FP,s2}=[(1-g_{FP})(1-\phi_{CATT-wb})+g_{FP}\phi_{GP}](1-\phi_{CATT1:4})\phi_{QBC}\phi_{LP-DC,s2}\phi_{WBC>20,s2}$

$\phi_{s2}=\phi'_{s2}+SS_{FP,s2}(1-\phi'_{s2})^{n}]$, where n=4

Accuracy of stage 1 classification:

$$\psi_{s1}=\psi_{s1,LP-DC}\psi_{s1,WBC>5}\frac{\phi_{s1}(L)}{\phi_{s1}}$$

Accuracy of stage 2 classification:

$$\psi_{s2}=\psi_{s2,LP-DC}+(1-\psi_{s2,LP-DC})\psi_{s2,WBC>5}\frac{\sigma_{s2}(L)}{\sigma_{s2}}+\frac{\sigma_{s2}(R)}{\sigma_{s2}}$$

**Kiri, Sudan (new algorithm)**

*[assuming QBC is used; otherwise, substitute QBC with CTC in all equations]*

Sensitivity of confirmation, if case is in stage 1 (no suspect follow-up):

*Left branch of algorithm:*

$$\sigma'_{s1}(L)=g_{TP}\sigma_{CATT-wb}\sigma_{GP}+[(1-g_{TP})\sigma_{CATT-wb}+g_{TP}\sigma_{CATT-wb}(1-\sigma_{GP})]\sigma_{CATT1:16}\sigma_{QBC}$$

*Right branch of algorithm:*

*Overall:*

$$\sigma'_{s1}=\sigma'_{s1}(L)+\sigma'_{s1}(R)$$

Sensitivity of confirmation, if case is in stage 2 (no suspect follow-up):

*Left branch of algorithm:*

$$\sigma'_{s2}(L)=\sigma'_{s1}(L)$$

*Right branch of algorithm:*

*Overall:*

$$\sigma'_{s2}=\sigma'_{s2}(L)+\sigma'_{s2}(R)$$

Sensitivity of confirmation, if case is in stage 1 (with suspect follow-up):

*[if village has ≥2% infection prevalence, the suspect is treated with pentamidine and not followed up]*

$\begin{matrix} SS_{TP,s1}=[(1-g_{TP})\sigma_{CATT-wb}+g_{TP}\sigma_{CATT-wb}(1-\sigma_{GP})]\sigma_{CATT1:16}(1-\sigma_{QBC})(1-\sigma_{LP-DC,s1}) \\ (1-\sigma_{WBC>20,s1}) \end{matrix}$

$\sigma_{s1}=\sigma'_{s1}+SS_{TP,s1}[1-(1-\sigma'_{s1})^{n}]$, where n=4

Sensitivity of confirmation, if case is in stage 2 (with suspect follow-up):

*[if village has ≥2% infection prevalence, the suspect is treated with pentamidine and not followed up]*

$\begin{matrix} SS_{TP,s2}=[(1-g_{TP})\sigma_{CATT-wb}+g_{TP}\sigma_{CATT-wb}(1-\sigma_{GP})]\sigma_{CATT1:16}(1-\sigma_{QBC})(1-\sigma_{LP-DC,s2}) \\ (1-\sigma_{WBC>20,s2}) \end{matrix}$

$\sigma_{s2}=\sigma'_{s2}+SS_{TP,s2}[1-(1-\sigma'_{s2})^{n}]$, where n=4

Specificity of confirmation, if case is in stage 1 (no suspect follow-up):

*Left branch of algorithm:*

$$\begin{matrix} \phi'_{s1}(L)=1-g_{FP}(1-\phi_{CATT-wb})(1-\phi_{GP})-[(1-g_{FP})(1-\phi_{CATT-wb})+g_{FP}(1-\phi_{CATT-wb})\phi_{GP}] \\ (1-\phi_{CATT1:16})(1-\phi_{QBC}) \end{matrix}$$

*Right branch of algorithm:*

$$\begin{matrix} \phi'_{s1}(R)=1-[(1-g_{FP})(1-\phi_{CATT-wb})+g_{FP}(1-\phi_{CATT-wb})\phi_{GP}](1-\phi_{CATT1:16})\phi_{QBC} \\ (1-\phi_{LP-DC,s1})-[(1-g_{FP})(1-\phi_{CATT-wb})+g_{FP}(1-\phi_{CATT-wb})\phi_{GP}](1-\phi_{CATT1:16})\phi_{QBC} \\ \phi_{LP-DC,s1}(1-\phi_{WBC>20,s1}) \end{matrix}$$

*Overall:*

$$\phi'_{s1}=\phi'_{s1}(L)+\phi'_{s1}(R)$$

Specificity of confirmation, if case is in stage 2 (no suspect follow-up):

*Left branch of algorithm:*

$$\phi'_{s2}(L)=\phi'_{s1}(L)$$

*Right branch of algorithm:*

$$\begin{matrix} \phi'_{s2}(R)=1-[(1-g_{FP})(1-\phi_{CATT-wb})+g_{FP}(1-\phi_{CATT-wb})\phi_{GP}](1-\phi_{CATT1:16})\phi_{QBC} \\ (1-\phi_{LP-DC,s2})-[(1-g_{FP})(1-\phi_{CATT-wb})+g_{FP}(1-\phi_{CATT-wb})\phi_{GP}](1-\phi_{CATT1:16})\phi_{QBC} \\ \phi_{LP-DC,s2}(1-\phi_{WBC>20,s2}) \end{matrix}$$

*Overall:*

$$\phi'_{s2}=\phi'_{s2}(L)+\phi'_{s2}(R)$$

Specificity of confirmation, if case is in stage 1 (with suspect follow-up):

$\begin{matrix} SS_{FP,s1}=[(1-g_{FP})(1-\phi_{CATT-wb})+g_{FP}(1-\phi_{CATT-wb})\phi_{GP}](1-\phi_{CATT1:16})\phi_{QBC} \\ \phi_{LP-DC,s1}\phi_{WBC>20,s1} \end{matrix}$

$\phi_{s1}=\phi'_{s1}+SS_{FP,s1}(1-\phi'_{s1})^{n}]$, where n=4

Specificity of confirmation, if case is in stage 2 (with suspect follow-up):

$\begin{matrix} SS_{FP,s2}=[(1-g_{FP})(1-\phi_{CATT-wb})+g_{FP}(1-\phi_{CATT-wb})\phi_{GP}](1-\phi_{CATT1:16})\phi_{QBC} \\ \phi_{LP-DC,s2}\phi_{WBC>20,s2} \end{matrix}$

$\phi_{s2}=\phi'_{s2}+SS_{FP,s2}(1-\phi'_{s2})^{n}]$, where n=4

Accuracy of stage 1 classification:

$$\psi_{s1}=\psi_{s1,LP-DC}\psi_{s1,WBC>5}\frac{\phi_{s1}(L)}{\phi_{s1}}$$

Accuracy of stage 2 classification:

$$\psi_{s2}=\psi_{s2,LP-DC}+(1-\psi_{s2,LP-DC})\psi_{s2,WBC>5}\frac{\sigma_{s2}(L)}{\sigma_{s2}}+\frac{\sigma_{s2}(R)}{\sigma_{s2}}$$

**Adjumani, Uganda**

Sensitivity of confirmation, if case is in stage 1 (no suspect follow-up):

*Left branch of algorithm:*

*Right branch of algorithm:*

$$\begin{matrix} \sigma'_{s1}(R)=[(1-g_{TP})\sigma_{CATT-wb}+g_{TP}\sigma_{CATT-wb}(1-\sigma_{GP})]\sigma_{CATT1:4}(1-\sigma_{CTC})(1-\sigma_{mAECT}) \\ \sigma_{LP-DC,s1}+[(1-g_{TP})\sigma_{CATT-wb}+g_{TP}\sigma_{CATT-wb}(1-\sigma_{GP})]\sigma_{CATT1:4}(1-\sigma_{CTC})(1-\sigma_{mAECT}) \\ (1-\sigma_{LP-DC,s1})\sigma_{WBC>20,s1} \end{matrix}$$

*Overall:*

$$\sigma'_{s1}=\sigma'_{s1}(L)+\sigma'_{s1}(R)$$

Sensitivity of confirmation, if case is in stage 2 (no suspect follow-up):

*Left branch of algorithm:*

$$\sigma'_{s2}(L)=\sigma'_{s1}(L)$$

*Right branch of algorithm:*

$$\begin{matrix} \sigma'_{s2}(R)=[(1-g_{TP})\sigma_{CATT-wb}+g_{TP}\sigma_{CATT-wb}(1-\sigma_{GP})]\sigma_{CATT1:4}(1-\sigma_{CTC})(1-\sigma_{mAECT}) \\ \sigma_{LP-DC,s2}+[(1-g_{TP})\sigma_{CATT-wb}+g_{TP}\sigma_{CATT-wb}(1-\sigma_{GP})]\sigma_{CATT1:4}(1-\sigma_{CTC})(1-\sigma_{mAECT}) \\ (1-\sigma_{LP-DC,s2})\sigma_{WBC>20,s2} \end{matrix}$$

*Overall:*

$$\sigma'_{s2}=\sigma'_{s2}(L)+\sigma'_{s2}(R)$$

Sensitivity of confirmation, if case is in stage 1 (with suspect follow-up):

$\begin{matrix} SS_{TP,s1}=[(1-g_{TP})\sigma_{CATT-wb}+g_{TP}\sigma_{CATT-wb}(1-\sigma_{GP})]\sigma_{CATT1:4}(1-\sigma_{CTC})(1-\sigma_{mAECT}) \\ (1-\sigma_{LP-DC,s1})(1-\sigma_{WBC>20,s1}) \end{matrix}$

$\sigma_{s1}=\sigma'_{s1}+SS_{TP,s1}[1-(1-\sigma'_{s1})^{n}]$, where n=1

Sensitivity of confirmation, if case is in stage 2 (with suspect follow-up):

$\begin{matrix} SS_{TP,s2}=[(1-g_{TP})\sigma_{CATT-wb}+g_{TP}\sigma_{CATT-wb}(1-\sigma_{GP})]\sigma_{CATT1:4}(1-\sigma_{CTC})(1-\sigma_{mAECT}) \\ (1-\sigma_{LP-DC,s2})(1-\sigma_{WBC>20,s2}) \end{matrix}$

$\sigma_{s2}=\sigma'_{s2}+SS_{TP,s2}[1-(1-\sigma'_{s2})^{n}]$, where n=1

Specificity of confirmation, if case is in stage 1 (no suspect follow-up):

*Left branch of algorithm:*

*Right branch of algorithm:*

*Overall:*

$$\phi'_{s1}=\phi'_{s1}(L)+\phi'_{s1}(R)$$

Specificity of confirmation, if case is in stage 2 (no suspect follow-up):

*Left branch of algorithm:*

$$\phi'_{s2}(L)=\phi'_{s1}(L)$$

*Right branch of algorithm:*

*Overall:*

$$\phi'_{s2}=\phi'_{s2}(L)+\phi'_{s2}(R)$$

Specificity of confirmation, if case is in stage 1 (with suspect follow-up):

$\begin{matrix} SS_{FP,s1}=[(1-g_{FP})(1-\phi_{CATT-wb})+g_{FP}(1-\phi_{CATT-wb})\phi_{GP}](1-\phi_{CATT1:4})\phi_{CTC}\phi_{mAECT}\phi_{LP-DC,s1} \\ \phi_{WBC>20,s1} \end{matrix}$

$\phi_{s1}=\phi'_{s1}+SS_{FP,s1}(1-\phi'_{s1})^{n}]$, where n=1

Specificity of confirmation, if case is in stage 2 (with suspect follow-up):

$\begin{matrix} SS_{FP,s2}=[(1-g_{FP})(1-\phi_{CATT-wb})+g_{FP}(1-\phi_{CATT-wb})\phi_{GP}](1-\phi_{CATT1:4})\phi_{CTC}\phi_{mAECT}\phi_{LP-DC,s2} \\ \phi_{WBC>20,s2} \end{matrix}$

$\phi_{s2}=\phi'_{s2}+SS_{FP,s2}(1-\phi'_{s2})^{n}]$, where n=1

Accuracy of stage 1 classification:

$$\psi_{s1}=\psi_{s1,LP-DC}\psi_{s1,WBC>5}\frac{\phi_{s1}(L)}{\phi_{s1}}$$

Accuracy of stage 2 classification:

$$\psi_{s2}=\psi_{s2,LP-DC}+(1-\psi_{s2,LP-DC})\psi_{s2,WBC>5}\frac{\sigma_{s2}(L)}{\sigma_{s2}}+\frac{\sigma_{s2}(R)}{\sigma_{s2}}$$

**Arua-Yumbe, Uganda**

Sensitivity of confirmation, if case is in stage 1 (no suspect follow-up):

*[as for Adjumani]*

Sensitivity of confirmation, if case is in stage 2 (no suspect follow-up):

*[as for Adjumani]*

Sensitivity of confirmation, if case is in stage 1 (with suspect follow-up):

$\begin{matrix} SS_{TP,s1}=[(1-g_{TP})\sigma_{CATT-wb}+g_{TP}\sigma_{CATT-wb}(1-\sigma_{GP})]\sigma_{CATT1:4}(1-\sigma_{CTC})(1-\sigma_{mAECT}) \\ (1-\sigma_{LP-DC,s1})(1-\sigma_{WBC>20,s1})\sigma_{CATT1:8} \end{matrix}$

$\sigma_{s1}=\sigma'_{s1}+SS_{TP,s1}[1-(1-\sigma'_{s1})^{n}]$, where n=4

Sensitivity of confirmation, if case is in stage 2 (with suspect follow-up):

$\begin{matrix} SS_{TP,s2}=[(1-g_{TP})\sigma_{CATT-wb}+g_{TP}\sigma_{CATT-wb}(1-\sigma_{GP})]\sigma_{CATT1:4}(1-\sigma_{CTC})(1-\sigma_{mAECT}) \\ (1-\sigma_{LP-DC,s2})(1-\sigma_{WBC>20,s2})\sigma_{CATT1:8} \end{matrix}$

$\sigma_{s2}=\sigma'_{s2}+SS_{TP,s2}[1-(1-\sigma'_{s2})^{n}]$, where n=4

Specificity of confirmation, if case is in stage 1 (no suspect follow-up):

*[as for Adjumani]*

Specificity of confirmation, if case is in stage 2 (no suspect follow-up):

*[as for Adjumani]*

Specificity of confirmation, if case is in stage 1 (with suspect follow-up):

$\begin{matrix} SS_{FP,s1}=[(1-g_{FP})(1-\phi_{CATT-wb})+g_{FP}(1-\phi_{CATT-wb})\phi_{GP}](1-\phi_{CATT1:4})\phi_{CTC}\phi_{mAECT}\phi_{LP-DC,s1} \\ \phi_{WBC>20,s1}(1-\phi_{CATT1:8}) \end{matrix}$

$\phi_{s1}=\phi'_{s1}+SS_{FP,s1}(1-\phi'_{s1})^{n}]$, where n=4

Specificity of confirmation, if case is in stage 2 (with suspect follow-up):

$\begin{matrix} SS_{FP,s2}=[(1-g_{FP})(1-\phi_{CATT-wb})+g_{FP}(1-\phi_{CATT-wb})\phi_{GP}](1-\phi_{CATT1:4})\phi_{CTC}\phi_{mAECT}\phi_{LP-DC,s2} \\ \phi_{WBC>20,s2}(1-\phi_{CATT1:8}) \end{matrix}$

$\phi_{s2}=\phi'_{s2}+SS_{FP,s2}(1-\phi'_{s2})^{n}]$, where n=4

Accuracy of stage 1 classification:

*[as for Adjumani]*

Accuracy of stage 2 classification:

*[as for Adjumani]*

**Reported sensitivity and specificity of HAT tests**

Table S1b. Estimates of sensitivity from the literature.

|  | **First author (year)** |  | **Estimate** |  | **Country (notes)** |
| --- | --- | --- | --- | --- | --- |
| **CATT-wb** | | | | | |
|  | Magnus (2002) [1] |  | 125/138 (90.6) |  | Uganda, Equatorial Guinea, DRC, Ivory Coast |
|  | Truc (2002) [2] |  | 65/66 (98.5) |  | Central African Republic, Ivory Coast |
|  | Noireau (1988) [3] |  | 127/154 (82.5) |  | Congo |
|  | Penchenier (2003) [4] |  | 16/16 (100.0) |  | Cameroon |
|  | Jamonneau (2000) [5] |  | 24/26 (92.3) |  | Ivory Coast |
|  | Enyaru (1998) [6] |  | 40/44 (90.9) |  | Uganda |
|  | Gastellu-Etchegorry (1987) [7] |  | 63/63 (100.0) |  | Uganda |
|  | Miezan (1991) [8] |  | 56/56 (100.0) |  | Ivory Coast |
| **CATT dilution 1:2** | | | | | |
|  | Paquet (1992) [9] |  | 191/193 (99.0) |  | Uganda |
|  | Magnus (1978) [10] |  | 53/54 (98.1) |  | DRC, Ivory Coast |
|  | Lutumba (2006) [11] |  | 152/154 (99.0) |  | DRC (approximated from Figure 2 in reference) |
| **CATT dilution 1:4** | | | | | |
|  | Paquet (1992) [9] |  | 186/193 (96.4) |  | Uganda |
|  | Magnus (1978) [10] |  | 52/54 (96.3) |  | DRC, Ivory Coast |
|  | Penchenier (2003) [4] |  | 58/59 (98.3) |  | Cameroon |
|  | Magnus (1991) [12] |  | 293/304 (96.4) |  | Congo, Equatorial Guinea, Ivory Coast, Sudan, DRC |
|  | Lutumba (2006) [11] |  | 142/154 (92.0) |  | DRC (approximated from Figure 2 in reference) |
| **CATT dilution 1:5** | | | | | |
|  | Gastellu-Etchegorry (1987) [7] |  | 63/63 (100.0) |  | Uganda |
|  | Miezan (1994) [13] |  | 55/57 (96.5) |  | Ivory Coast |
| **CATT dilution 1:8** | | | | | |
|  | Paquet (1992) [9] |  | 174/193 (90.2) |  | Uganda, CATTwb+, ELISA+ or parasite+ |
|  | Magnus (1978) [10] |  | 49/54 (90.7) |  | DRC, Ivory Coast |
|  | Magnus (1991) [12] |  | 272/304 (89.5) |  | Congo, Equatorial Guinea, Ivory Coast, Sudan, DRC |
|  | Lutumba (2006) [11] |  | 121/154 (78.8) |  | DRC |
| **CATT dilution 1:10** | | | | | |
|  | Gastellu-Etchegorry (1987) [7] |  | 45/63 (71.4) |  | Uganda |
|  | Miezan (1994) [13] |  | 48/57 (84.2) |  | Ivory Coast |
| **CATT dilution 1:16** | | | | | |
|  | Paquet (1992) [9] |  | 144/193 (74.6) |  | Uganda |
|  | Magnus (1978) [10] |  | 33/54 (61.1) |  | DRC, Ivory Coast |
|  | Magnus (1991) [12] |  | 172/304 (56.6) |  | Congo, Equatorial Guinea, Ivory Coast, Sudan, DRC |
|  | Lutumba (2006) [11] |  | 72/154 (46.5) |  | DRC (approximated from Figure 2 in reference) |
| **CATT dilution 1:20** | | | | | |
|  | Gastellu-Etchegorry (1987) [7] |  | 33/63 (52.4) |  | Uganda |
|  | Miezan (1994) [13] |  | 29/57 (50.9) |  | Ivory Coast |
| **CATT dilution 1:32** | | | | | |
|  | Paquet (1992) [9] |  | 75/193 (38.9) |  | Uganda |
|  | Magnus (1978) [10] |  | 14/54 (25.9) |  | DRC, Ivory Coast |
|  | Magnus (1991) [12] |  | 52/304 (17.1) |  | Congo, Equatorial Guinea, Ivory Coast, Sudan, DRC |
|  | Lutumba (2006) [11] |  | 28/154 (18.0) |  | DRC (approximated from Figure 2 in reference) |
| **CATT dilution 1:40** | | | | | |
|  | Gastellu-Etchegorry (1987) [7] |  | 23/63 (36.5) |  | Uganda |
|  | Miezan (1994) [13] |  | 14/57 (24.6) |  | Ivory Coast |
| **CATT dilution 1:64** | | | | | |
|  | Paquet (1992) [9] |  | 15/193 (7.8) |  | Uganda |
|  | Magnus (1978) [10] |  | 1/54 (1.9) |  | DRC, Ivory Coast |
|  | Magnus (1991) [12] |  | 11/304 (3.6) |  | Congo, Equatorial Guinea, Ivory Coast, Sudan, DRC |
| **CATT dilution 1:80** | | | | | |
|  | Gastellu-Etchegorry (1987) [7] |  | 19/63 (30.2) |  | Uganda |
|  | Miezan (1994) [13] |  | 2/57 (3.5) |  | Ivory Coast |
| **CATT dilution 1:128** | | | | | |
|  | Magnus (1991) [12] |  | 2/304 (0.7) |  | Congo, Equatorial Guinea, Ivory Coast, Sudan, DRC |
| **CATT dilution 1:160** | | | | | |
|  | Gastellu-Etchegorry (1987) [7] |  | 7/63 (11.1) |  | Uganda |
|  | Miezan (1994) [13] |  | 0/57 (0.0) |  | Ivory Coast |
| **CATT dilution 1:320** | | | | | |
|  | Gastellu-Etchegorry (1987) [7] |  | 5/63 (7.9) |  | Uganda |
| **GP** | | | | | |
|  | Lutumba (2006) [11] |  | 29/52 (55.8) |  | DRC |
|  | Bailey (1992) [14] |  | 19/30 (63.3) |  | Uganda |
|  | Miezan (1994) [13] |  | 34/58 (58.6) |  | Ivory Coast |
|  | Truc (1994) [15] |  | 7/11 (63.6) |  | Ivory Coast |
| **CTC** | | | | | |
|  | Lutumba (2006) [11] |  | 87/154 (56.5) |  | DRC |
|  | Lumsden (1981) [16] |  | 24/33 (72.7) |  | Ivory Coast |
|  | Miezan (1994) [13] |  | 28/58 (48.3) |  | Ivory Coast |
|  | Truc (1994) [15] |  | 6/11 (54.5) |  | Ivory Coast |
| **mAECT** | | | | | |
|  | Lutumba (2006) [11] |  | 116/154 (75.3) |  | DRC |
|  | Miezan (1994) [13] |  | 49/58 (84.5) |  | Ivory Coast |
|  | Truc (1994) [15] |  | 10/11 (90.9) |  | Ivory Coast |
| **QBC** | | | | | |
|  | Truc (1994) [15] |  | 11/11 (100.0) |  | Ivory Coast |
| **CSF-DC (if case is in stage 1)** | | | | | |
|  | Miezan (1998) [17] |  | 0/19 (0.0) |  | Ivory Coast |
|  | Miezan (1994) [13] |  | 0/8 (0.0) |  | Ivory Coast |
|  | Kyambadde (2000) [18] |  | 0/13 (0.0) |  | Uganda |
|  | Jamonneau (2003) [19] |  | 1/37 (2.7) |  | Ivory Coast |
| * | Lejon (2003) [20] |  | 20/75 (26.7) |  | Ivory Coast, Uganda |
| **CSF-DC (if case is in stage 2)** | | | | | |
|  | Miezan (1998) [17] |  | 42/71 (59.2) |  | Ivory Coast |
|  | Miezan (1994) [13] |  | 40/50 (80.0) |  | Ivory Coast |
|  | Kyambadde (2000) [18] |  | 13/21 (61.9) |  | Uganda |
|  | Jamonneau (2003) [19] |  | 24/36 (66.7) |  | Ivory Coast |
|  | Lejon (2003) [20] |  | 137/197 (69.5) |  | Ivory Coast, Uganda |
| **WBC density >20/μL (if case is in stage 1)** | | | | | |
|  | Miezan (1998) [17] |  | 2/19 (10.5) |  | Ivory Coast |
|  | Lejon (2003) [20] |  | 2/75 (2.7) |  | Ivory Coast, Uganda |
|  | Jamonneau (2003) [19] |  | 3/37 (8.1) |  | Ivory Coast |
|  | Bisser (2002) [21] |  | 1/41 (2.4) |  | Congo |
| **WBC density >20/μL (if case is in stage 2)** | | | | | |
|  | Miezan (1998) [17] |  | 46/71 (64.8) |  | Ivory Coast |
|  | Lejon (2003) [20] |  | 137/197 (69.5) |  | Ivory Coast, Uganda |
|  | Jamonneau (2003) [19] |  | 22/36 (61.1) |  | Ivory Coast |
|  | Bisser (2002) [21] |  | 21/54 (38.9) |  | Congo |

* only included in conservative analysis.

Table S1c. Estimates of specificity from the literature.

|  | **First author (year)** |  | **Estimate** |  | **Country (notes)** |
| --- | --- | --- | --- | --- | --- |
| **CATT-wb** | | | | | |
|  | Magnus (2002) [1] |  | 350/358 (97.8) |  | from non-HAT endemic areas |
| * | Magnus (2002) [1] |  | 2143/2170 (98.8) |  | Uganda, Equatorial Guinea, DRC, Ivory Coast |
| * | Truc (2002) [2] |  | 832/874 (95.2) |  | Central African Republic, Ivory Coast |
| * | Noireau (1988) [3] |  | 133/141 (94.3) |  | Congo (infected with Loa loa, M. persistens) |
| * | Penchenier (2003) [4] |  | 4943/5239 (94.4) |  | Cameroon |
| * | Jamonneau (2000) [5] |  | 393/425 (92.5) |  | Ivory Coast |
|  | Bafort (1986) [22] |  | 172/179 (96.1) |  | South Africa (outpatients) |
|  | Bafort (1986) [22] |  | 62/63 (98.4) |  | South Africa (patients with various parasitic diseases) |
| * | Gastellu-Etchegorry (1987) [7] |  | 44/50 (88.0) |  | Uganda (inpatients) |
| * | Miezan (1991) [8] |  | 385/401 (96.0) |  | Ivory Coast |
| * | Paquet (1992) [9] |  | 164/222 (73.9) |  | Uganda (inpatients) |
| **CATT dilution 1:2 (among CATT-wb positives only)** | | | | | |
|  | Paquet (1992) [9] |  | 2/58 (3.4) |  | Uganda (inpatients) |
|  | Lutumba (2006) [11] |  | 41/282 (14.5) |  | DRC (approximated from Figure 2 in reference) |
| **CATT dilution 1:4 (among CATT-wb positives only)** | | | | | |
|  | Paquet (1992) [9] |  | 31/58 (53.4) |  | Uganda (inpatients) |
|  | Lutumba (2006) [11] |  | 104/282 (37.0) |  | DRC (approximated from Figure 2 in reference) |
| **CATT dilution 1:8 (among CATT-wb positives only)** | | | | | |
|  | Paquet (1992) [9] |  | 48/58 (82.8) |  | Uganda (inpatients) |
|  | Lutumba (2006) [11] |  | 165/282 (58.5) |  | DRC |
| **CATT dilution 1:16 (among CATT-wb positives only)** | | | | | |
|  | Paquet (1992) [9] |  | 56/58 (96.6) |  | Uganda (inpatients) |
|  | Lutumba (2006) [11] |  | 224/282 (79.5) |  | DRC (approximated from Figure 2 in reference) |
| **CATT dilution 1:32 (among CATT-wb positives only)** | | | | | |
|  | Paquet (1992) [9] |  | 57/58 (98.3) |  | Uganda (inpatients) |
|  | Lutumba (2006) [11] |  | 260/282 (92) |  | DRC (approximated from Figure 2 in reference) |
| **CATT dilution 1:64 (among CATT-wb positives only)** | | | | | |
|  | Paquet (1992) [9] |  | 58/58 (100.0) |  | Uganda (inpatients) |
| **GP** | | | | | |
|  | [none] |  |  |  |  |
| **CTC** | | | | | |
|  | Kyambadde (2000) [18] |  | 15/15 (100.0) |  | Uganda |
| **mAECT** | | | | | |
|  | [none] |  |  |  |  |
| **QBC** | | | | | |
|  | [none] |  |  |  |  |
| **CSF-DC** | | | | | |
|  | Miezan (1998) [17] |  | 19/19 (100.0) |  | Ivory Coast |
|  | Miezan (1994) [13] |  | 8/8 (100.0) |  | Ivory Coast |
|  | Kyambadde (2000) [18] |  | 13/13 (100.0) |  | Uganda |
|  | Jamonneau (2003) [19] |  | 36/37 (97.3) |  | Ivory Coast |
| * | Lejon (2003) [20] |  | 55/75 (73.3) |  | Ivory Coast, Uganda |
| **WBC density >20/μL** | | | | | |
|  | Miezan (1998) [17] |  | 17/19 (89.5) |  | Ivory Coast |
|  | Lejon (2003) [20] |  | 73/75 (97.3) |  | Ivory Coast, Uganda |
|  | Jamonneau (2003) [19] |  | 34/37 (91.9) |  | Ivory Coast |
|  | Bisser (2002) [21] |  | 40/41 (97.6) |  | Congo |

* only included in conservative analysis.

Table S1d. Estimates of staging accuracy from the literature.

|  | **First author (year)** |  | **Proportion of cases correctly classified as stage 1 (%)** |  | **Proportion of cases correctly classified as stage 2 (%)** |  | **Country** |
| --- | --- | --- | --- | --- | --- | --- | --- |
| **CSF-DC** | | | | | | | |
|  | Miezan (1998) [17] |  | 19/19 (100.0) |  | 42/71 (59.2) |  | Ivory Coast |
|  | Kyambadde (2000) [18] |  | 13/13 (100.0) |  | 13/21 (61.9) |  | Uganda |
|  | Jamonneau (2003) [19] |  | 36/37 (97.3) |  | 24/36 (66.7) |  | Ivory Coast |
|  | Lejon (2003) [20] |  | 55/75 (73.3) * |  | 137/197 (69.5) |  | Ivory Coast, Uganda |
|  | Miezan (1994) [13] |  | 8/8 (100.0) |  | 40/50 (80.0) |  | Ivory Coast |
| **WBC density >5/μL** | | | | | | | |
|  | Miezan (1998) [17] |  | 11/19 (57.9) |  | 58/71 (81.7) |  | Ivory Coast |
|  | Kyambadde (2000) [18] |  | 10/13 (76.9) |  | 17/21 (81.0) |  | Uganda |
|  | Jamonneau (2003) [19] |  | 25/37 (67.6) |  | 27/36 (75.0) |  | Ivory Coast |
|  | Bisser (2002) [21] |  | 30/41 (73.2) |  | 38/54 (70.4) |  | Congo |
|  | Cattand (1988) [23] |  | n/a |  | 28/51 (54.9) |  | Ivory Coast |
| **WBC density >10/μL** | | | | | | | |
|  | Miezan (1998) [17] |  | 14/19 (73.7) |  | 51/71 (71.8) |  | Ivory Coast |
|  | Bisser (2002) [21] |  | 38/41 (92.7) |  | 27/54 (50.0) |  | Congo |
| **WBC density >20/μL** | | | | | | | |
|  | Miezan (1998) [17] |  | 17/19 (89.5) |  | 46/71 (64.8) |  | Ivory Coast |
|  | Lejon (2003) [20] |  | 73/75 (97.3) |  | 137/197 (69.5) |  | Ivory Coast, Uganda |
|  | Jamonneau (2003) [19] |  | 34/37 (91.9) |  | 22/36 (61.1) |  | Ivory Coast |
|  | Bisser (2002) [21] |  | 40/41 (97.6) |  | 21/54 (38.9) |  | Congo |

* only included in conservative analysis.

**Fitted polynomial functions for the accuracy of CATT dilutions**

Figure S1a. Fitted and reported values of the sensitivity of CATT dilutions among CATT-wb positive individuals.

Each grey circle represents one published estimate of sensitivity for a given CATT dilution. The size of each circle is proportional to the sample size on which the estimate is based (larger studies are attributed more weight in the fitting procedure). The blue dotted line represents the fitted polynomial function.

Figure S1b. Fitted and reported values of the specificity of CATT dilutions among CATT-wb positive individuals.

Each grey circle represents one published estimate of specificity for a given CATT dilution. The size of each circle is proportional to the sample size on which the estimate is based (larger studies are attributed more weight in the fitting procedure). The blue dotted line represents the fitted polynomial function.

**References**

1. Magnus E, Lejon V, Bayon D, Buyse D, Simarro P, et al. (2002) Evaluation of an EDTA version of CATT/Trypanosoma brucei gambiense for serological screening of human blood samples. Acta Trop 81: 7-12.

2. Truc P, Lejon V, Magnus E, Jamonneau V, Nangouma A, et al. (2002) Evaluation of the micro-CATT, CATT/Trypanosoma brucei gambiense, and LATEX/T b gambiense methods for serodiagnosis and surveillance of human African trypanosomiasis in West and Central Africa. Bull World Health Organ 80: 882-886.

3. Noireau F, Lemesre JL, Nzoukoudi MY, Louembet MT, Gouteux JP, et al. (1988) Serodiagnosis of sleeping sickness in the Republic of the Congo: comparison of indirect immunofluorescent antibody test and card agglutination test. Trans R Soc Trop Med Hyg 82: 237-240.

4. Penchenier L, Grebaut P, Njokou F, Eboo Eyenga V, Buscher P (2003) Evaluation of LATEX/T.b.gambiense for mass screening of Trypanosoma brucei gambiense sleeping sickness in Central Africa. Acta Trop 85: 31-37.

5. Jamonneau V, Truc P, Garcia A, Magnus E, Buscher P (2000) Preliminary evaluation of LATEX/T. b. gambiense and alternative versions of CATT/T. b. gambiense for the serodiagnosis of human african trypanosomiasis of a population at risk in Cote d'Ivoire: considerations for mass-screening. Acta Trop 76: 175-183.

6. Enyaru JC, Matovu E, Akol M, Sebikali C, Kyambadde J, et al. (1998) Parasitological detection of Trypanosoma brucei gambiense in serologically negative sleeping-sickness suspects from north-western Uganda. Ann Trop Med Parasitol 92: 845-850.

7. Gastellu Etchegorry M, Godin C, Fievet N, Aquadro B (1987) Study about the specificity and sensitivity of the CATT. Medecins Sans Frontieres.

8. Miezan T, Doua F, Cattand P, de Raadt P (1991) [Evaluation of Testryp CATT applied to blood samples on filter paper and on diluted blood in a focus of trypanosomiasis due to Trypanosoma brucei gambiense in the Ivory Coast]. Bull World Health Organ 69: 603-606.

9. Paquet C (1992) Depistage de la trypanosomiase en Ouganda: strategie d'utilisation du test d'agglutination sur carte (CATT *T.b. gambiense*). Bordeaux: Universite de Bordeaux II.

10. Magnus E, Vervoort T, Van Meirvenne N (1978) A card-agglutination test with stained trypanosomes (C.A.T.T.) for the serological diagnosis of T. B. gambiense trypanosomiasis. Ann Soc Belg Med Trop 58: 169-176.

11. Lutumba P, Robays J, Miaka C, Kande V, Mumba D, et al. (2006) [Validity, cost and feasibility of the mAECT and CTC confirmation tests after diagnosis of African of sleeping sickness]. Trop Med Int Health 11: 470-478.

12. Magnus E, Vervoort T, Van Meirvenne N (1991) Laboratory evaluation of the card agglutination test (CATT) for serodiagnosis of sleeping sickness due to *T.b. gambiense*. Institute of Tropical Medicine, Antwerp, Belgium.

13. Miezan TW, Meda AH, Doua F, Cattand P (1994) [Evaluation of the parasitologic technics used in the diagnosis of human Trypanosoma gambiense trypanosomiasis in the Ivory Coast]. Bull Soc Pathol Exot 87: 101-104.

14. Bailey JW, Smith DH (1992) The use of the acridine orange QBC technique in the diagnosis of African trypanosomiasis. Trans R Soc Trop Med Hyg 86: 630.

15. Truc P, Bailey JW, Doua F, Laveissiere C, Godfrey DG (1994) A comparison of parasitological methods for the diagnosis of gambian trypanosomiasis in an area of low endemicity in Cote d'Ivoire. Trans R Soc Trop Med Hyg 88: 419-421.

16. Lumsden WH, Kimber CD, Dukes P, Haller L, Stanghellini A, et al. (1981) Field diagnosis of sleeping sickness in the Ivory Coast. I. Comparison of the miniature anion-exchange/centrifugation technique with other protozoological methods. Trans R Soc Trop Med Hyg 75: 242-250.

17. Miezan TW, Meda HA, Doua F, Yapo FB, Baltz T (1998) Assessment of central nervous system involvement in gambiense trypanosomiasis: value of the cerebro-spinal white cell count. Trop Med Int Health 3: 571-575.

18. Kyambadde JW, Enyaru JC, Matovu E, Odiit M, Carasco JF (2000) Detection of trypanosomes in suspected sleeping sickness patients in Uganda using the polymerase chain reaction. Bull World Health Organ 78: 119-124.

19. Jamonneau V, Solano P, Garcia A, Lejon V, Dje N, et al. (2003) Stage determination and therapeutic decision in human African trypanosomiasis: value of polymerase chain reaction and immunoglobulin M quantification on the cerebrospinal fluid of sleeping sickness patients in Cote d'Ivoire. Trop Med Int Health 8: 589-594.

20. Lejon V, Reiber H, Legros D, Dje N, Magnus E, et al. (2003) Intrathecal immune response pattern for improved diagnosis of central nervous system involvement in trypanosomiasis. J Infect Dis 187: 1475-1483.

21. Bisser S, Lejon V, Preux PM, Bouteille B, Stanghellini A, et al. (2002) Blood-cerebrospinal fluid barrier and intrathecal immunoglobulins compared to field diagnosis of central nervous system involvement in sleeping sickness. J Neurol Sci 193: 127-135.

22. Bafort JM, Schutte CH, Gathiram V (1986) Specificity of the Testryp CATT card agglutination test in a non-sleeping-sickness area of Africa. S Afr Med J 69: 541-542.

23. Cattand P, Miezan BT, de Raadt P (1988) Human African trypanosomiasis: use of double centrifugation of cerebrospinal fluid to detect trypanosomes. Bull World Health Organ 66: 83-86.
